# Experimental and Mechanistic Validation of PARP1pred for Identifying Potent Leads

**DOI:** 10.34133/csbj.0152

**Published:** 2026-07-07

**Authors:** Sermsiri Chitphuk, Wasana Stitchantrakul, Rakkreat Wikiniyadhanee, Donniphat Dejsuphong, Kanchanok Kodchakorn, Tassanee Lerksuthirat

**Affiliations:** ^1^ Research Center, Faculty of Medicine Ramathibodi Hospital, Mahidol University, Bangkok 10400, Thailand.; ^2^ Program in Translational Medicine, Chakri Naruebodindra Medical Institute, Faculty of Medicine Ramathibodi Hospital, Mahidol University, Samut Prakan 10540, Thailand.; ^3^ Computational Simulation and Modelling Laboratory (CSML), Department of Chemistry, Faculty of Science, Chiang Mai University, Chiang Mai 50200, Thailand.

## Abstract

Poly(adenosine diphosphate-ribose) polymerase 1 (PARP1) is a pivotal target for treating homologous recombination-deficient cancers through the mechanism of synthetic lethality. While machine learning has accelerated the identification of novel inhibitors, many models lack experimental validation and high-resolution mechanistic insights. In this study, we evaluated the predictive robustness of the PARP1pred model using a hierarchical pipeline. Initial bioactivity predictions for candidates in unseen chemical space were validated through biochemical and cellular sensitivity assays using a panel of isogenic TK6 cell lines. Subsequently, molecular docking, 100-ns molecular dynamics simulations, and molecular mechanics Poisson–Boltzmann surface area (MM-PBSA) energetic analysis were performed to provide a structural and thermodynamic rationale for the observed inhibitory potencies. The workflow successfully identified ZINC49069486 as a highly potent nanomolar lead that induced selective synthetic lethality in BRCA1-deficient cells. Crucially, the pipeline correctly classified ZINC67913374 as biologically inactive [median inhibitory concentration (IC_50_) > 100 μM], successfully identifying a false positive previously proposed in the literature as a potential inhibitor. MM-PBSA analysis revealed that the inactivity of ZINC67913374 was driven by an excessive polar solvation penalty that outweighed its favorable docking score. While ZINC98208034 and ZINC8793749 showed moderate enzymatic inhibition, they failed to induce a synthetic lethality response. These results confirm that the PARP1pred-driven hierarchical framework effectively prioritizes experimentally validated lead compound while filtering out deceptive computational hits, providing an accessible and robust strategy for PARP1 inhibitor discovery.

## Graphical Abstract

**Figure:**
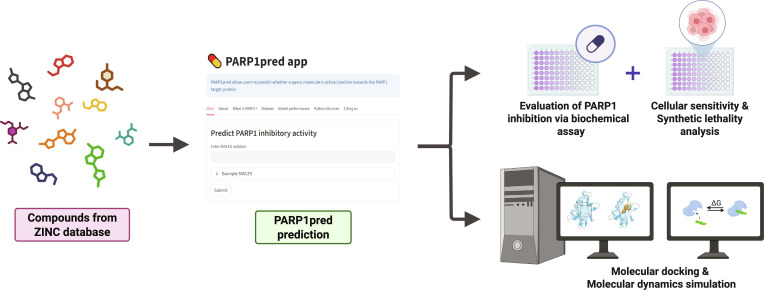

## Introduction

Poly(adenosine diphosphate-ribose) polymerase 1 (PARP1) is a crucial enzyme in the detection and repair of DNA damage, serving as a primary guardian of genomic stability [1–3]. It functions predominantly through the regulation of 2 critical repair pathways: base excision repair (BER) and the alternative end-joining pathway [4,5]. While tightly regulated under physiological conditions, the dysfunction or overactivation of PARP1 is implicated in the pathogenesis of various conditions, including neurodegenerative [6], inflammatory [7], and cardiovascular diseases [8]. Most notably, PARP1 has emerged as a cornerstone in oncology through the mechanism of synthetic lethality, where its inhibition selectively induces cell death in homologous recombination (HR)-deficient cancers [9]. Consequently, the development of targeted PARP1 inhibitors has become an increasingly effective and established therapeutic strategy for treating HR-deficient malignancies [10,11].

Although several PARP inhibitors (PARPis) are U.S. Food and Drug Administration (FDA)-approved as standard-of-care regimens, their clinical utility remains limited by hematologic toxicity, acquired resistance, and constraints on combination dosing [12–14]. Because synthetic lethality in these tumors depends predominantly on PARP1, whereas PARP2 is dispensable for efficacy but crucial for hematopoietic stem-cell survival, next-generation PARP1-selective inhibitors aim to preserve or enhance antitumor activity while reducing toxicities, improving pharmacokinetics (including central nervous system penetration), and expanding combination strategies.

The clinical demand for novel PARP1 inhibitors has catalyzed the integration of advanced computational frameworks into the drug discovery process. Computational approaches have revolutionized the field by enabling the design, optimization, and prediction of drug behavior in silico, substantially reducing the time and cost associated with traditional experimental methods [15]. These approaches aid in target identification, drug design, virtual screening, toxicity prediction, and personalized medicine, making drug discovery more efficient, cost-effective, and precise [16]. As computational power increases and machine learning (ML) techniques evolve, their integration into drug discovery will continue to expand, potentially leading to faster development of more effective and safer therapeutics [17].

Numerous studies have utilized these in silico workflows to identify novel PARP1 inhibitors. Li et al. [18] employed computational screening of natural compounds from the ZINC database to a single candidate as a potential PARP1 inhibitor through molecular docking and molecular dynamics (MD) simulations. However, its efficacy was not validated in subsequent in vitro screening. Shi et al. [19] utilized ligand-binding crystal structures combined with an in silico screening workflow, based on structure-guided molecular docking and subsequent MD simulations, to discover novel antitumor compounds targeting PARP1. Two compounds identified through an in vitro PARP1 enzymatic assay were further shown to exhibit antitumor activity in human colorectal carcinoma (HCT-116) cell lines. Demuth et al. [20] identified novel PARP inhibitors from the ComPlat library through molecular docking. Two promising candidates demonstrated potent PARP1 inhibition, as validated by in vitro PARP activity measurements and colorectal cancer cell-based assays.

More recently, researchers have shifted toward ML approaches to develop predictive models for PARP1 activity. Aldakheel et al. [21] built models using the BindingDB dataset, selecting a random forest model to screen phytochemicals for PARP1 inhibition. The candidates were further evaluated for drug-likeness using Lipinski’s Rule of Five and mechanistic analysis via molecular docking and MD simulations. Our previous study utilized bioactivity data from the ChEMBL database to develop an ML predictive model, which was deployed as a web server to provide public access for predicting PARP1 inhibition. However, that model has not yet been experimentally validated in vitro, nor has it been subject to further exploration of its mechanistic details using structure-based simulations [22].

Building upon these findings, the present study aims to evaluate the predictive utility and generalizability of the PARP1pred ML model and refine the design framework for next-generation candidates. To address the persistent challenges in in silico lead identification and bridge the gap between computational hits, biochemical inhibition, and functional cellular efficacy, we utilized a hierarchical workflow to assess a set of compounds, specifically excluding those used in the model’s initial training, focusing on natural candidates from the ZINC database. By integrating high-resolution MD simulations and molecular mechanics Poisson–Boltzmann surface area (MM-PBSA) energetic analysis with biochemical and cellular sensitivity assays, we aim to provide a structural and thermodynamic rationale for inhibitory potency. This approach seeks to refine the development of novel PARP1 inhibitors and provide a robust framework for identifying preclinical-grade leads while filtering out computationally deceptive false positives.

## Materials and Methods

### Compound selection and preparation

Five compounds were identified from the ZINC database [23] and selected for this study based on their commercial availability: 4 natural candidates (ZINC98208034, ZINC67913374, α-mangostin, and ZINC8793749) and 1 synthetic compound, ZINC49069486. ZINC98208034, ZINC67913374, and ZINC8793749 were purchased from Molport (Riga, Latvia), while olaparib (an FDA-approved PARP inhibitor used as a positive control) and ZINC49069486 were purchased from MedChemExpress (NJ, USA). α-Mangostin (Carbosynth Limited, Berkshire, UK) was included as a negative control. Despite its broad biological activities, it has no known PARP1 inhibitory effect [24,25], allowing us to verify that our experimental cascade accurately distinguishes targeted PARP1 inhibition from nonspecific assay interference. ZINC67913374 was of particular interest, as it was previously identified as a potential PARP1 inhibitor through molecular docking and dynamics simulations [18]. Notably, with the exception of olaparib, none of the compounds in this study were part of the curated dataset used to develop the PARP1pred model in our previous work [22]. All chemical compounds were dissolved in 100% (v/v) dimethyl sulfoxide (DMSO) to a final stock concentration of 10 mM.

### Bioactivity prediction using PARP1pred

The ZINC and ChEMBL identification numbers, canonical SMILES representations, PARP1pred predictions, and 2-dimensional (2D) structures of these compounds are presented in Table 1. Candidate bioactivity was predicted using the PARP1pred web application [22]. Chemical structures in canonical SMILES format were converted into 881-bit PubChem fingerprints using the PaDEL-Descriptor software. These fingerprints served as input for a random forest classification model, which was trained on 2,018 nonredundant compounds curated from the ChEMBL database. The model categorized compounds as active [median inhibitory concentration (IC_50_) ≤ 1 μM] or inactive (IC_50_ ≥ 10 μM) based on structural features prioritized by the Gini index.

**Table 1.** ZINC identification, canonical SMILES, chemical structure, and PARP1pred bioactivity predictions for the candidate compounds

Compound	Canonical SMILES	Chemical structure	PARP1pred
Olaparib	O=C(c1cc(Cc2n[nH]c(=O)c3ccccc23)ccc1F)N1CCN(C(=O)C2CC2)CC1	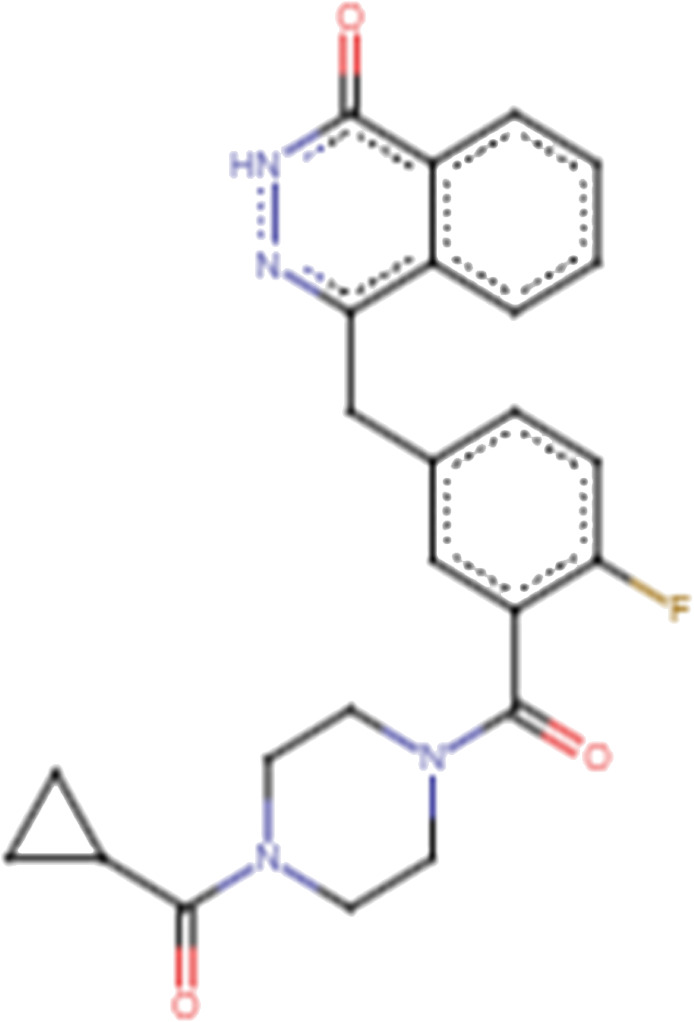	Active
ZINC49069486	NC(=O)c1cccc2nc(-c3ccc([C@@H]4CCCN4)cc3F)[nH]c12	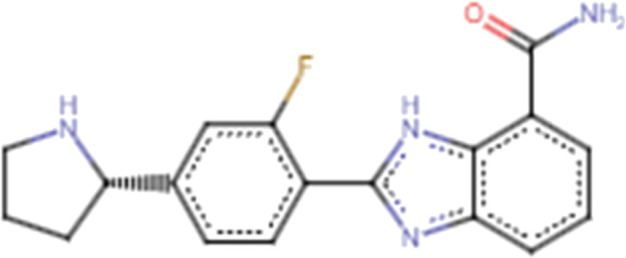	Active
ZINC98208034	O=C(CCc1nc2ccccc2c(=O)[nH]1)N[C@H](CO)Cc1ccccc1	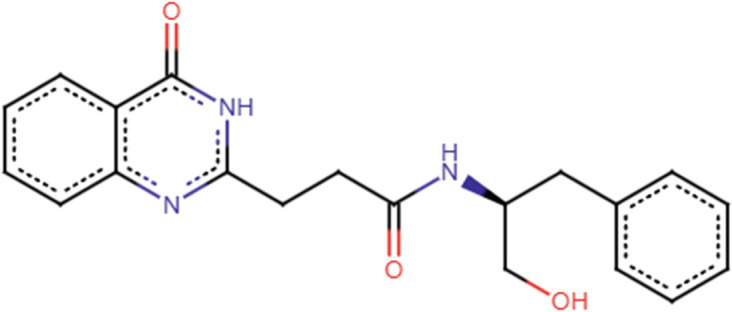	Active
ZINC67913374	OC[C@H]1O[C@@H](O[C@H](CCCCc2ccc(O)cc2)CCc2ccc(O)cc2)[C@H](O[C@@H]2OC[C@](O)(CO)[C@H]2O)[C@@H](O)[C@@H]1O	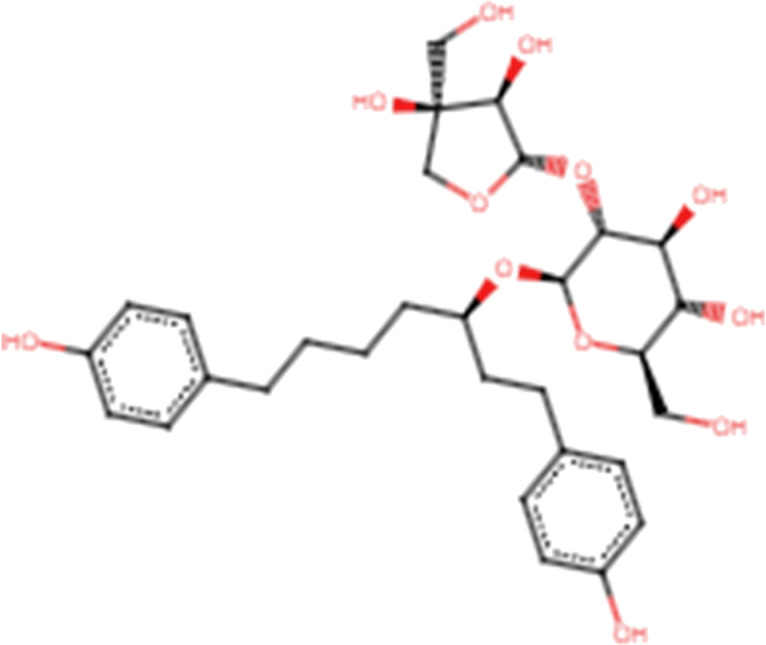	Inactive
ɑ-Mangostin	COc1c(O)cc2oc3cc(O)c(CC=C(C)C)c(O)c3c(=O)c2c1CC=C(C)C	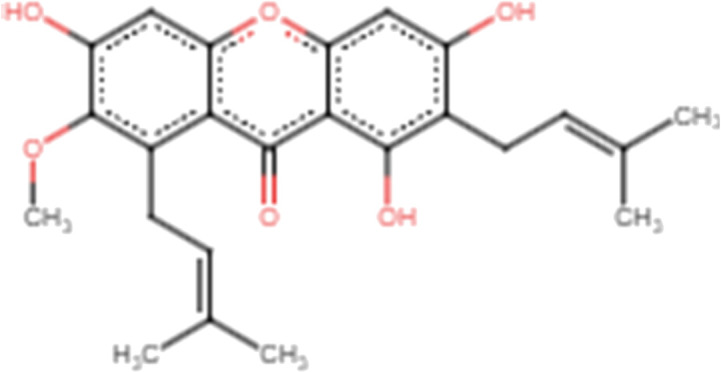	Inactive
ZINC8793749	Cc1nc2ccccc2c(=O)[nH]1	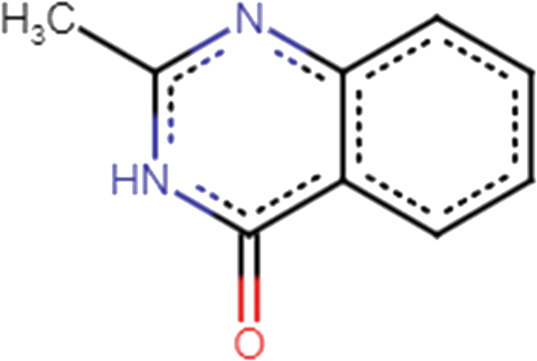	Inactive

### PARP inhibition assay

The PARP inhibitory assay was performed according to the manufacturer’s protocol of the PARP Universal Colorimetric Assay Kit (R&D Systems, MN, US). Briefly, the precoated histone was rehydrated with 1× PARP buffer and incubated at room temperature for 30 min. Next, various concentrations of the inhibitor in 1× PARP buffer were added, followed by the diluted PARP enzyme at 0.25 units/well. 1× PARP cocktail was then added, and the mixture was incubated further at room temperature for 60 min. The wells were washed, and diluted streptavidin–horseradish peroxidase was added and incubated at room temperature for 60 min. Subsequently, prewarmed TACS-Sapphire colorimetric substrate was added and incubated in the dark at room temperature for 15 min. The reaction was stopped using 0.2 M HCl (Merck, NJ, USA). Absorbance at 450 nm was measured using an Infinite 200Pro microplate reader (Tecan, Männedorf, Switzerland). The inhibition percentages were determined by normalizing the absorbance signal to that of the well containing no inhibitor. The resulting percentages were then plotted, and the IC_50_ was calculated using GraphPad Prism software, version 9.5.1 (GraphPad Software Inc., MA, USA). The experiments were performed on 3 technical replicates.

### Cell culture condition

Human lymphoblastoid TK6-derived wild-type (WT), PARP1 knockout (*PARP1^−/−^*), and breast cancer type 1 susceptibility protein conditional knockout (*BRCA1^AID/AID^*) human cell lines [26,27] were maintained in RPMI 1640 medium (Gibco, MA, USA) supplemented with 5% horse serum (Invitrogen, MA, USA), 200 μg/ml sodium pyruvate (Sigma-Alderich, MO, USA), and 100 U/ml penicillin/streptomycin (Gibco, MA, USA). The cultures were incubated at 37 °C in a humidified environment with 5% CO_2_ and were used for experiments when they reached 70% to 80% confluences. *BRCA1^AID/AID^* TK6 cells were induced for BRCA1 protein degradation by culturing in media containing 250 μM auxin (Sigma-Aldrich, MO, USA). This induction began with a 2-h pretreatment period before drug exposure and was maintained throughout the experiment to inhibit BRCA1 function for the cell survival assay [28].

### Cellular sensitivity assay

For the PARP inhibitor sensitivity assay, human TK6 suspension cells were seeded in 96-well clear assay plates (Corning, NY, USA) at 30,000 cells/ml (6,000 cells/well) for WT and *PARP1^−/−^*, and 100,000 cells/ml (20,000 cells/well) for *BRCA1^AID/AID^* in 200 μl of medium per well*.* Cells were treated with various concentrations of tested compounds for a duration of 72 h at 37 °C. Cells viability were assessed using the CellTiter-Glo Luminescent Cell Viability Assay, following the manufacturer’s protocol (Promega, WI, USA). Briefly, 50 μl of each cell sample was mixed with 50 μl of CellTiter-Glo reagent. After 10 min at room temperature, the luminescence of the mixture was measured using Infinite 200Pro microplate reader (Tecan, Männedorf, Switzerland). Survival percentages were determined by normalizing the luminescent signal to that of untreated cells. These percentages were then plotted, and the IC_50_ values were analyzed using GraphPad Prism software, version 9.5.1 (GraphPad Software Inc.). The experiments were performed on 3 biological replicates.

### Structure-based computational calculations

#### Protein–ligand preparation

The 3D PARP1 protein was obtained from the Protein Data Bank (PDB code: 4UND) as a monomer structure [29]. This experimentally determined x-ray crystal structure was selected because it contains a structurally resolved PARP1 catalytic domain and inhibitor-binding pocket, providing a validated template for structure-based calculations. Consequently, additional protein structure prediction using AlphaFold or other machine learning-based approaches was not performed, as experimentally resolved structures are generally preferred when high-quality crystallographic data are available. To prepare the target protein structure for docking, charges and polar hydrogen atoms were added using the prepare_receptor4.py script from MGLTools 1.5.6 [30].

The 3D structures of all compounds were retrieved from the ZINC database (in the structure data file format) [23] and subjected to a short energy minimization using Amber 22 [31]. The minimization converged when the root mean squared (RMS) gradient tolerance reached 0.0100 kcal/mol Å. Subsequently, individual PDB files for docking were prepared using the prepare_ligand4.py script from MGLTools [30], retaining only the largest nonbonded fragment present.

#### Molecular docking

Molecular docking was performed using AutoDock Vina to anchor the compounds into the potential binding site of the PARP1 protein [32]. Default parameter settings were used for the docking process [33]. A grid box, with dimensions (*x*, *y*, *z*) of 40 × 40 × 40 Å and a point spacing of 0.375 Å, was defined to encompass the catalytic pocket of the human PARP1 crystal structure (PDB: 4UND) [29].

The dimension of the grid box was set to cover residues of the active site of the target protein. The exhaustiveness parameter, which dictates the amount of conformational sampling effort, was set to 200, with an energy range of 10 kcal/mol. The maximum number of poses to be reported was set to 20, utilizing the built-in clustering analysis with a 2.0-Å cutoff.

The purpose of molecular docking in this study was to identify plausible binding poses and evaluate whether candidate compounds could occupy the canonical PARP1 catalytic pocket. Docking results were not used as standalone evidence of inhibitory activity but rather served as the structural starting point for subsequent MD simulations and energetic analyses.

#### MD simulations

Docked PARP1–ligand complexes from AutoDock Vina with the highest binding affinities were selected based on both their predicted binding affinities and the structural alignment of the docked ligand poses with the reference inhibitor-binding configuration within the catalytic pocket for MD simulations [34]. Each complex system under periodic boundary condition was solvated in a cubic box of TIP3P water model extending to 10 Å along each direction from the complex model with counter ions added to neutralize the system. LeaP module embedded in AMBER 22 [31] was used for adding the missing atoms with the FF14SB force field [35] for applying the description of the protein characterization. The cutoff distance was kept to 12 Å in order to compute the nonbonded interactions.

The CUDA-based AMBER 22 program was used by the PMEMD.CUDA module [36] for speeding up the simulation times. Each system was first minimized using the steepest descent protocol followed by a conjugate gradient procedure for relaxation and elimination of overlapping atoms. The first step was to allow, out of 10,000 iterations, only water molecules to move. In the second step, each of the 10,000 iterations, hydrogen and protein side chains were relaxed, in a fixed order. Finally, 20,000 steps were calculated with the restriction-free system. After energy minimization, the optimized MD systems were carried out gradually heating (H) phase NVT (constant number of atoms, volume, and temperature) ensemble with the fixed protein atoms for 100 ps from 0 to 310.15 K by using a force constant of 10 kcal/mol Å^−2^. This was followed by 1,000 ps of equilibration (Eq1) phase NVT-MD at 310.15 K at a force constant of 5.0 kcal/mol Å^−2^. Then, 10,000 ps without any constrained forces of equilibration-2 (Eq2) phase NPT-MD were performed for each fully flexible equilibrium system at the same temperature and 1 atm pressure. The density of each system was about 1.0 g/cm^3^. Finally, 100 ns of unrestrained production (Prod) phase NVT-MD simulation were applied at a constant temperature of 310.15 K. The time step of 2 fs was set, and the trajectory was recorded every 0.2 ps. Structural analysis of the root mean square deviation (RMSD) and the principal components analysis (PCA) was carried out by CPPTRAJ module [37] using the Amber 22 program. The structural images were presented using BIOVIA Discovery Studio Client (BIOVIA, Dassault Systèmes, Discovery Studio, v20, San Diego: Dassault Systèmes, 2019).

#### Binding free energy and per-residue decomposition energy analysis

The final 30-ns trajectory contained 15,000 saved frames. To minimize temporal autocorrelation between adjacent trajectory frames, 300 decorrelated snapshots extracted at 100-ps intervals were used for Amber MM-PBSA calculations [38] to estimate the binding free energy (Δ*G*_binding_, kcal/mol) of the complex. The binding free energy (Δ*G*_binding_, kcal/mol) and per-residue decomposition energy (Δ*G*_decomp_, kcal/mol) were calculated for each variant species in the energetic framework of the MM-PBSA method, and the free energy was computed from the following equation:

$$\begin{array}{c} \begin{array}{c} \Delta G_{\text{binding}}=\Delta G_{\text{Complex}}-\left(\Delta G_{\text{PARP1}}+\Delta G_{\text{Ligand}}\right)\\ \;=\Delta E_{\mathrm{vdW}}+\Delta E_{\mathrm{EEL}}+\Delta G_{\text{solvation}} \end{array} \end{array}$$ (1)

where Δ*E*_vdW_ and Δ*E*_EEL_ refer to the van der Waals and electrostatic contribution calculated by the molecular mechanics force field, and Δ*G*_solvation_ (Δ*G*_PS_ + Δ*G*_Nonpolar_) represents the solvation free energy that consists of polar solvation (Δ*G*_PS_) and nonpolar solvation (Δ*G*_Nonpolar_) free energies.

#### Principal components analysis

To evaluate the displacement of atoms and conformational dynamics of a protein complex, PCA was performed and analyzed using a covariance matrix-based approach [34,39,40]. The elements of the positional covariance matrix C were obtained based on Eq. (2):

$$C_{ij}=\langle \left(x_{i}-\langle x_{i}\rangle \right)\;\times \;\left(x_{j}-\langle x_{j}\rangle \right)\rangle$$ (2)

where $x_{i}$ and $x_{j}$ are the instant coordinates of the *i*th and *j*th Cα atoms of the systems for use in building the covariance matrix *C*, while 〈$x_{i}$〉 and 〈$x_{j}$〉 refer to an ensemble average. The averaged values are computed over the Prod-MD simulations after superimposition on a reference structure using the CPPTRAJ module of AMBER 22, and solvent water molecules and neutralizing ions added by the Leap module are stripped prior to MD trajectory generation. PCA was performed for Cα atoms on 15,000 snapshots each. PC1 and PC2, which represent the first 2 PCs, are created from the trajectories averaged from the WT and the variant systems. The trajectories were analyzed for the relative motions about their center of masses.

### Statistical analysis

Statistical analysis was performed using GraphPad Prism software version 9.5.1 (GraphPad Software Inc.). All results were expressed as averages and SDs.

## Results

### In silico prediction of PARP1 bioactivity using PARP1pred

The predicted biological activities of the 6 candidate compounds, selected to evaluate the model’s performance on novel chemical scaffolds not included in the original curated dataset, are summarized in Table 1. The PARP1pred identified 3 compounds (olaparib, ZINC49069486, and ZINC98208034) as active, based on their structural features and predicted inhibitory potential. Conversely, ZINC67913374, α-mangostin, and ZINC8793749 were classified as inactive. The PubChem fingerprints generated via PARP1pred are provided in Tables S1 and S2, alongside the top 20 fingerprints prioritized by Gini index (Table S3). The analysis demonstrates a positive correlation between predicted bioactivity and the prevalence of these high-importance substructural features; active candidates consistently exhibited a greater frequency of these top-tier fingerprints than their inactive counterparts.

### Evaluation of PARP1 inhibition via biochemical assay

**Fig. 1.**
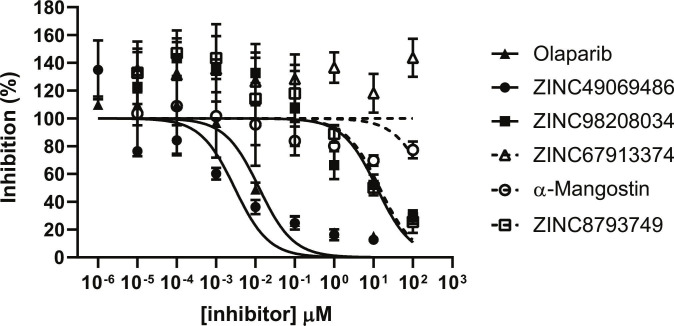
In vitro inhibitory profiles of evaluated compounds against recombinant human PARP1. The graph illustrates the dose-dependent percentage of inhibition of histone PARylation for each candidate compound. Data were obtained using a biochemical assay. The concentration ranges tested were 0 to 10 μM for olaparib and ZINC49069486, and 0 to 100 μM for the remaining compounds. Each data point represents the mean ± SD from 3 technical replicates.

To validate the computational predictions, the inhibitory potency of the selected compounds was evaluated using a biochemical assay. This assay measured the ability of each compound to inhibit the PARylation of histone proteins by recombinant human PARP1 (Fig. 1). The resulting IC_50_ values, detailed in Table 2, allowed the compounds to be categorized into 3 distinct potency groups based on their inhibitory thresholds: <1 μM, 1 to 100 μM, and >100 μM.

**Table 2.** IC_50_ profiles from the PARP inhibitory assay in response to various inhibitors. IC_50_ values are presented as the mean ± SD.

Compounds	IC_50_ (μM)
Olaparib	0.012 ± 0.001
ZINC49069486	0.003 ± 0.001
ZINC98208034	12.238 ± 1.808
ZINC67913374	>100
ɑ-Mangostin	>100
ZINC8793749	15.791 ± 4.053

The first group, characterized by sub-micromolar potency, included olaparib (IC_50_ = 0.012 ± 0.001 μM) and ZINC49069486 (IC_50_ = 0.003 ± 0.001 μM). The second group (1 to 100 μM) comprised ZINC98208034 (IC_50_ = 12.238 ± 1.808 μM) and ZINC8793749 (IC_50_ = 15.791 ± 4.053 μM). Finally, ZINC67913374 and ɑ-mangostin exhibited the lowest inhibitory activity, with IC_50_ values exceeding 100 μM.

### Cellular sensitivity and synthetic lethality analysis

**Fig. 2.**
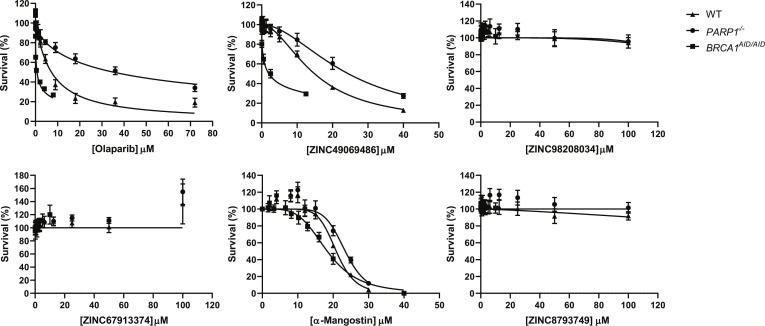
Cellular survival assay of each evaluated compound in TK6 cell lines. The percentage of cell survival is shown for each compound at various concentrations in the WT, *PARP1^−/−^*, and *BRCA1^AID/AID^* cell lines. Inhibitor concentrations range from 0 to 100 μM for WT and *PARP1^−/−^*, and from 0 to 40 μM for *BRCA1^AID/AID^*.

To evaluate the compounds within a complex biological environment, we conducted cellular sensitivity assays using a panel of isogenic TK6 cell lines (WT, *PARP1^−/−^*, and *BRCA1^AID/AID^*). This approach allowed us to determine whether the biochemical inhibition observed in the assay translated into a cellular response, specifically assessing the compounds’ ability to induce synthetic lethality in BRCA1-deficient cells (Fig. 2). As expected, the FDA-approved drug olaparib exhibited a characteristic sensitivity profile: *PARP1^−/−^* > WT > *BRCA1^AID/AID^* (Table 3; IC_50_ values of 35.590 ± 3.732, 7.548 ± 1.453, and 0.697 ± 0.165 μM, respectively). This profile confirms PARP1-dependent cellular activity and its ability to induce synthetic lethality in BRCA1-deficient cells.

**Table 3.** IC_50_ profiles from the sensitivity assay in response to various inhibitors. IC_50_ values are presented as the mean ± SD.

Compounds	IC_50_ (μM)		
	WT	*PARP1^−/−^*	*BRCA1* ^*AID/AID*^
Olaparib	7.548 ± 1.453	35.590 ± 3.732	0.697 ± 0.165
ZINC49069486	14.867 ± 0.701	24.460 ± 1.562	2.072 ± 0.585
ZINC98208034	>100	>100	>10
ZINC67913374	>100	>100	>10
ɑ-Mangostin	20.865 ± 0.219	23.515 ± 0.771	18.330 ± 0.806
ZINC8793749	>100	>100	>10

ZINC49069486 and ɑ-mangostin displayed similar sensitivity trends to olaparib. For ZINC49069486, the IC_50_ values were 24.460 ± 1.562, 14.867 ± 0.701, and 2.072 ± 0.585 μM for *PARP1^−/−^*, WT, and *BRCA1^AID/AID^* cells, respectively. In contrast, ɑ-mangostin yielded higher IC_50_ values (23.515 ± 0.771, 20.865 ± 0.219, and 18.330 ± 0.806 μM). Notably, the fold change in IC_50_ between *PARP1^−/−^* and *BRCA1^AID/AID^* was 50-fold for olaparib, 12-fold for ZINC49069486, and only 1.2-fold for ɑ-mangostin. These data suggest that while ɑ-mangostin impacts cell viability, it lacks the PARP1-specific hypersensitivity observed in the other compounds. Finally, ZINC98208034, ZINC67913374, and ZINC8793749 exhibited comparable dose–response profiles, with IC_50_ values exceeding 100 μM in both WT and *PARP1^−/−^*, and remaining above 10 μM in *BRCA1^AID/AID^*. These results indicate that these 3 natural compounds possess negligible cytotoxicity under the tested conditions.

### Computational studies

#### Molecular docking study

To evaluate whether the 6 candidate compounds could access and occupy the canonical PARP1 (hPARP1) inhibitor-binding pocket, molecular docking was performed as an initial structural assessment. Docking was intended to characterize potential binding modes and residue contacts rather than to establish biological activity or inhibitory potency. The docking results revealed that the compounds share a common binding site with olaparib, although they exhibit slightly different binding configurations (Fig. S1).

Among the evaluated systems, olaparib exhibited the strongest predicted binding affinity toward hPARP1 (−12.4 kcal/mol; Table 4), supporting its role as a clinically validated PARP inhibitor. The binding pose of olaparib was stabilized by extensive interactions with residues located within the conserved binding cleft, including key aromatic and catalytic pocket residues such as W861, H862, Y889, Y896, F897, and Y907, together with polar residues D766, S864, R865, and N868 [41,42]. These residues are consistent with the reported active site environment of PARP1 inhibitors, suggesting that olaparib forms an optimized binding configuration that tightly anchors the ligand within the catalytic domain.

**Table 4.** Binding affinity (kcal/mol) and the common binding residues in the PARP1–ligand complexes by AutoDock Vina

Complexes	Binding affinity	Common residues within 3.5 Å
Olaparib	−12.4	E763, D766, D770, W861, H862, G863, S864, R865, N868, I872, G876, L877, R878, A880, Y889, G894, Y896, F897, A898, K903, S904, Y907, N987, E988, Y989
ZINC49069486	−10.6	Q759, A762, E763, D766, W861, H862, G863, T887, G888, Y889, M890, Y896, F897, A898, K903, S904, Y907, N987, E988, Y989
ZINC98208034	−9.5	E763, D766, H862, G863, S864, Y889, M890, Y896, F897, A898, K903, S904, Y907, E988
ZINC67913374	−9.8	E763, D766, N767, L769, H862, G863, S864, R865, N868, I872, R878, I879, A880, P881, Y889, M890, G894, I895, Y896, F897, A898, K903, S904, Y907, H909, E988
α-Mangostin	−8.9	D766, D770, H862, S864, N868, I872, L877, R878, I879, A880, G888, Y889, M890, Y896, F897, A898, K903, S904, Y907, E988
ZINC8793749	−7.2	W861, H862, G863, S864, Y896, F897, A898, K903, S904, Y907, N987, E988

Within the candidate inhibitors, ZINC49069486 showed the highest docking affinity (−10.6 kcal/mol), ranking closest to olaparib and suggesting strong binding complementarity within the same pocket. ZINC49069486 shares multiple common binding residues with olaparib, including E763, D766, W861, H862, G863, Y889, Y896, F897, A898, K903, S904, Y907, N987, E988, and Y989. This overlap indicates that ZINC49069486 may adopt an olaparib-like binding orientation and potentially retains critical contacts required for competitive inhibition within the binding cleft. Importantly, the presence of both aromatic residues (W861, Y889, Y896, F897) and polar residues (D766, H862, S904) suggests a combined stabilization mechanism through hydrophobic packing and hydrogen-bonding networks.

ZINC67913374 also demonstrated a favorable docking score (−9.8 kcal/mol), slightly stronger than ZINC98208034 (−9.5 kcal/mol). The predicted binding mode of ZINC67913374 involved a broad interaction network, including residues E763, D766, H862, G863, S864, R865, N868, I872, R878, A880, Y889, M890, G894, Y896, F897, A898, K903, S904, Y907, and E988. The engagement of both charged residues (R865, R878) and aromatic residues (Y889, Y896, F897) suggests that ZINC67913374 may form an extended interaction pattern that enhances its docking affinity. Similarly, ZINC98208034 displayed a comparable binding profile by interacting with key catalytic pocket residues including E763, D766, H862, G863, S864, Y889, M890, Y896, F897, A898, K903, S904, Y907, and E988, indicating that ZINC98208034 can occupy the same functional site but possibly with reduced contact diversity compared to ZINC67913374.

In contrast, α-mangostin and ZINC8793749 exhibited lower docking affinities (−8.9 and −7.2 kcal/mol, respectively), suggesting weaker predicted stabilization within the binding cleft. Although α-mangostin still maintained interactions with several conserved residues such as D766, H862, S864, N868, Y889, Y896, F897, K903, S904, Y907, and E988, the reduced docking score may reflect less optimal steric complementarity or weaker polar contact formation compared with olaparib. For ZINC8793749, the limited binding affinity corresponds to fewer interacting residues, mainly W861, H862, G863, S864, Y896, F897, A898, K903, S904, Y907, N987, and E988, suggesting a less extensive interaction network and potentially weaker binding confinement within the catalytic pocket.

Overall, docking analysis revealed that all candidate ligands were capable of occupying the canonical PARP1 inhibitor-binding site and shared substantial overlap with residues that define the olaparib binding pocket. Among the evaluated compounds, ZINC49069486 exhibited the highest docking affinity among the candidate ligands and showed extensive overlap with key residues involved in olaparib binding, suggesting that it can adopt a comparable binding orientation within the catalytic pocket. Similarly, ZINC67913374 and ZINC98208034 displayed favorable docking scores and maintained interactions with conserved active site residues, indicating that these compounds can also access the canonical inhibitor-binding region. In contrast, α-mangostin and particularly ZINC8793749 exhibited lower docking affinities and fewer favorable contacts within the catalytic pocket. Importantly, despite these differences, the docking results alone were insufficient to distinguish biologically active compounds from inactive candidates. For example, ZINC67913374 exhibited a favorable docking score and extensive interactions with catalytic site residues but subsequently demonstrated negligible inhibitory activity in both biochemical and cellular assays. These findings highlight that docking primarily evaluates geometric complementarity and potential pocket occupancy within a largely static protein structure, but does not fully account for protein flexibility, solvent reorganization, or long-timescale conformational effects. Consequently, compounds exhibiting favorable docking scores may still display unfavorable thermodynamic behavior during subsequent MD simulations. Therefore, docking analysis in the present study was used primarily to identify plausible binding poses and provide structural starting points for subsequent MD simulations and MM-PBSA calculations rather than as definitive evidence of binding affinity or inhibitor potency.

#### MD simulations study

##### Structural stability and flexibility of hPARP1 binding complexes

**Fig. 3.**
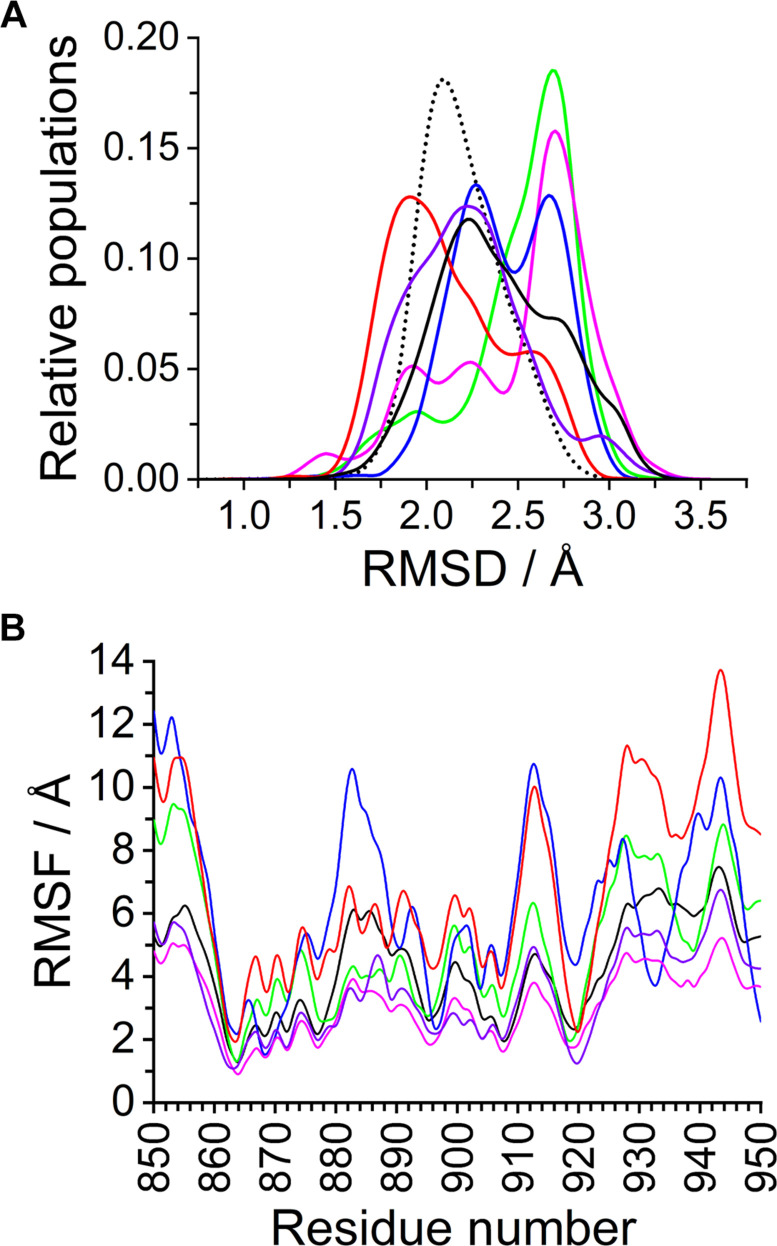
Structural analysis of each hPARP1 in complex system analyzed during Prod-MD simulations. (A) RMSD population distributions of hPARP1 in the Apo state (black dotted line) and in complex with olaparib (black solid line), ZINC49069486 (green solid line), ZINC98208034 (blue solid line), ZINC67913374 (magenta solid line), ɑ-mangostin (purple solid line), and ZINC8793749 (red solid line). (B) Detailed RMSF profile of the catalytic pocket region (residues 850 to 920) of hPARP1. The displayed region contains several key ligand-binding residues involved in PARP1 inhibition, including H862, Y889, I895, Y896, K903, S904, and Y907. The full-length RMSF profile for all residues is provided in Fig. S3.

To extensively sample the conformational space and refine the molecular geometries, the 3D complexes of each hPARP1–ligand system obtained from AutoDock Vina were subjected to MD simulations. These 100-ns simulations were performed for both the apo-hPARP1 protein and the 6 hPARP1–ligand complexes. To assess the structural dynamics and stability of the complexes, the root mean square deviations (RMSDs) of the protein–ligand complex relative to their initial optimized structures was monitored throughout the simulation trajectory, as depicted in Fig. 3. The RMSD plots revealed steady oscillations and minimal fluctuations for each complex model compared to the apo-system (Fig. S2). These RMSD profiles quantify the degree of conformational deviation from the starting structure, where lower RMSD values generally indicate superior structural stability.

As illustrated in the RMSD distribution profiles in Fig. 3A, the RMSD distribution analysis was performed to evaluate the conformational stability and structural flexibility of hPARP1 in the unbound (apo) state and in complex with olaparib and the 5 candidate ligands during the production MD simulations. The apo system exhibited a single dominant RMSD population centered at approximately ~2.2 Å with a moderately broad distribution spanning ~1.5 to 3.0 Å, indicating that hPARP1 maintains an overall stable fold in the absence of ligand but retains intrinsic flexibility and dynamic structural fluctuations. This apo RMSD profile represents the baseline conformational behavior of the protein, reflecting natural breathing motions and conformational adjustments occurring during the simulation.

In the ligand-bound systems, distinct differences in RMSD population profiles were observed, suggesting ligand-dependent modulation of hPARP1 stability. Among all complexes, ZINC49069486 displayed the narrowest and sharpest RMSD distribution with a dominant peak around ~2.6 to 2.7 Å, indicating that ZINC49069486 binding strongly restricts conformational variability and stabilizes hPARP1 into a highly rigid structural ensemble. This narrow RMSD population suggests that the ZINC49069486-bound complex predominantly occupies a single conformational basin throughout the simulation, reflecting enhanced structural stability and a well-defined binding mode. In contrast, ZINC98208034 exhibited a clear bimodal RMSD distribution with 2 major peaks around ~2.2 Å and ~2.7 Å, implying that the protein samples 2 major conformational states during the simulation. Such bimodal behavior suggests conformational switching in the binding environment and indicates increased flexibility compared to ZINC49069486, consistent with a less rigid but still structurally stable protein–ligand interaction.

Interestingly, ZINC67913374 demonstrated a dominant sharp peak near ~2.75 Å but also displayed multiple additional shoulders and minor subpopulations at lower RMSD values (~1.4 to 2.3 Å). This multimodal pattern indicates that although the ZINC67913374 complex adopts a major stable conformational state, it simultaneously samples several alternative substates, reflecting increased conformational heterogeneity. Therefore, ZINC67913374 appears to stabilize hPARP1 partially but does not fully restrict the protein dynamics, suggesting that the binding pocket may undergo dynamic rearrangements and adopt multiple binding-associated conformations. In the case of α-mangostin, the RMSD distribution was relatively broad and flattened, with a main peak around ~2.0 to 2.2 Å and an extended tail reaching ~3.1 Å. This broad distribution indicates that α-mangostin binding allows greater structural fluctuations and conformational diversity compared to Apo and other ligand-bound systems, suggesting reduced rigidity and weaker stabilization of the protein structure within the binding environment. Such a profile may reflect an unstable or less optimized binding mode that fails to strongly confine the motion of the catalytic region.

For ZINC8793749, the RMSD distribution showed a main population around ~1.8 Å with a secondary shoulder near ~2.4 to 2.6 Å, indicating the presence of at least 2 conformational states during the simulation. Although the RMSD values remain within a moderate range (~1.3 to 2.8 Å), the 2-state distribution suggests dynamic conformational transitions, implying that ZINC8793749 binding stabilizes the protein fold but still permits conformational adaptation of the binding pocket.

In addition to the candidate ligands, the olaparib-bound system was included as a positive control because olaparib is a well-established FDA-approved PARP1 inhibitor with experimentally validated binding activity. The RMSD distribution of the olaparib complex shows a broader and more continuous population (centered approximately around ~2.3 to 2.5 Å) compared to the ZINC49069486 complex, which displays a sharper and narrower RMSD peak (~2.6 to 2.7 Å). This indicates that olaparib maintains a stable binding interaction while still allowing moderate flexibility in the protein structure, reflecting the balance between ligand-induced stabilization and natural conformational breathing of the binding pocket.

Overall, comparison of RMSD distributions indicates that ligand binding markedly modulates the conformational stability and flexibility of hPARP1. Notably, the olaparib-bound complex, used here as a positive control and clinically approved PARP inhibitor, exhibits a relatively stable RMSD population centered around ~2.3 to 2.5 Å with a moderately broad distribution, suggesting that olaparib maintains a well-balanced binding mode that stabilizes the protein while still allowing limited intrinsic flexibility required for natural structural breathing motions. In comparison, ZINC49069486 shows a narrower and sharper RMSD distribution than olaparib, implying even greater rigidity and structural stabilization of the complex, which may reflect strong confinement of the binding pocket. Meanwhile, in terms of RMSD population distributions, ZINC98208034, and ZINC8793749 exhibited broader and/or multimodal profiles than olaparib, indicating greater conformational heterogeneity during the simulations. In contrast, α-mangostin demonstrates the broadest RMSD distribution, indicating the highest flexibility and least stable conformational restriction compared to both Apo and olaparib systems. Taken together, these results highlight that while olaparib provides a stable and experimentally validated binding reference profile, ZINC49069486 appears to induce stronger structural rigidity than the commercial inhibitor, whereas the other candidates show weaker stabilization effects, potentially corresponding to less persistent binding interactions and reduced binding affinity.

To explore the structural analysis of the potential model in sufficient detail, we calculated the dynamics of the residues in terms of the analysis of the root mean square fluctuations (RMSFs), which provides residue-level insights into the local flexibility of hPARP1 upon ligand binding, reflecting how each compound influences structural stability of the binding environment and adjacent loop regions. Overall, as seen in Fig. 3B, the detailed catalytic pocket RMSF profiles (residues 850 to 920) indicate that ligand binding markedly modulates the dynamic behavior of hPARP1, particularly within the catalytic ART domain [29,43]. Several pronounced fluctuation peaks were observed, mainly distributed within the region spanning approximately residues ~650 to 1,000 (full RMSF profile; Fig. S3), which corresponds to the C-terminal catalytic domain containing the nicotinamide adenine dinucleotide (NAD^+^)/olaparib binding pocket [44]. Importantly, the key binding pocket residues reported in crystallographic studies of PARP1–olaparib complexes, including H862, G863, Y896, S904, Y907, and E988, are located within this dynamic region, supporting the relevance of RMSF changes to ligand-induced stabilization of the functional active site.

In the olaparib-bound complex, RMSF values remain moderate across most residues, indicating that olaparib preserves overall structural stability while allowing limited flexibility in surrounding loop regions. This controlled dynamic behavior is consistent with its established role as a clinically effective PARP inhibitor, stabilizing the active site architecture without imposing excessive rigidity. Notably, the binding pocket region (~860 to 910) shows restrained fluctuations, supporting a stable binding mode with maintained conformational adaptability.

For the ZINC49069486 complex, the RMSF profile is broadly comparable to olaparib, although slightly higher mobility is observed in several segments. This suggests moderate stabilization of the catalytic domain while permitting increased flexibility in loop regions adjacent to the binding site. In contrast, ZINC98208034 exhibits the highest RMSF peaks, particularly around residues ~700 to 780, reflecting pronounced loop mobility and structural destabilization. Such elevated fluctuations may indicate weaker confinement of the catalytic environment and reduced stability of the binding pocket architecture.

Conversely, ZINC67913374 and α-mangostin show consistently lower RMSF values across the catalytic domain, suggesting strong restriction of residue mobility and enhanced structural rigidity. These reduced fluctuations imply that both ligands promote a stabilized binding environment and may effectively anchor key loop regions surrounding the active site. Meanwhile, ZINC8793749 displays elevated RMSF across multiple regions, especially above residue ~900, indicating increased flexibility and dynamic instability near the binding pocket, which may reduce binding persistence.

Overall, the RMSF results suggest that ZINC67913374 and α-mangostin provide the greatest reduction in residue mobility on hPARP1 flexibility, while ZINC98208034 and ZINC8793749 induce the highest structural mobility and potential destabilization. The ZINC49069486 complex shows an RMSF pattern largely comparable to olaparib but with slightly increased flexibility in several loop segments, indicating moderate stabilization of the catalytic domain while still allowing greater local mobility than the reference drug. In comparison, olaparib maintains an intermediate but well-balanced flexibility profile, supporting its role as a reliable positive control that stabilizes the binding pocket while preserving necessary dynamic features of the catalytic domain.

##### PCA projection analysis of hPARP1 apo and ligand-bound complexes

**Fig. 4.**
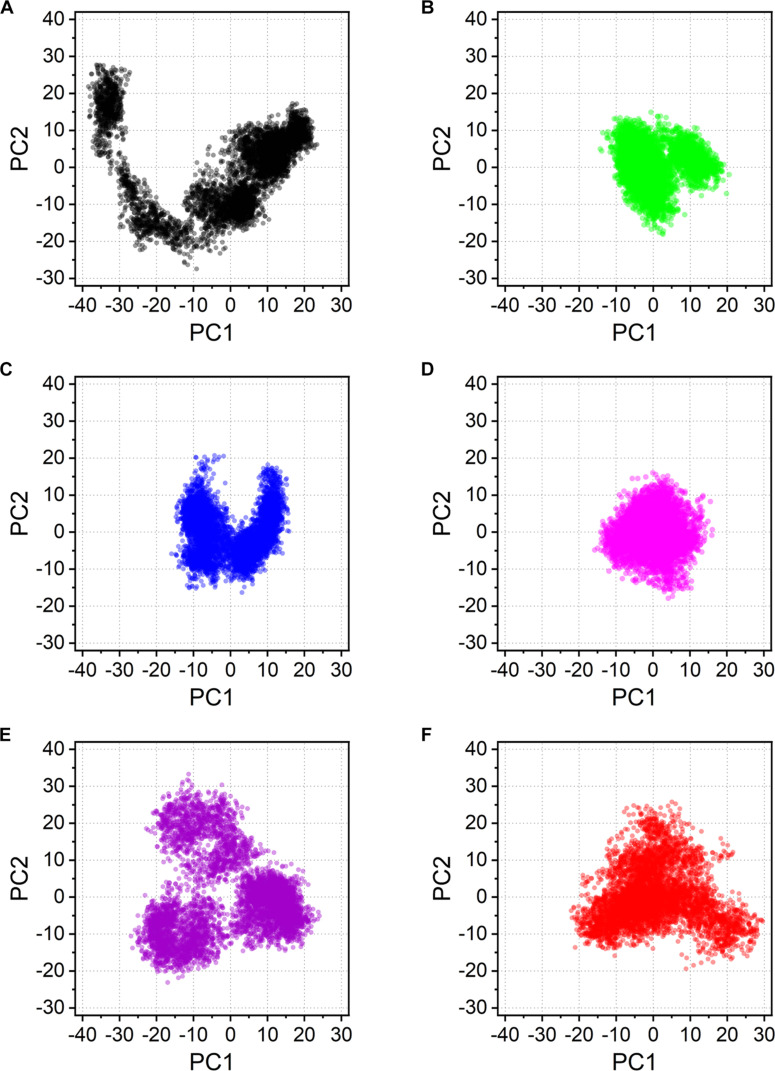
PCA projection of Cα-atom motions in the hPARP1 system. PCA profiles were generated by plotting the first 2 PCs (PC1 and PC2) in the conformational space for the hPARP1 complexed with olaparib (A, black), ZINC49069486 (B, green), ZINC98208034 (C, blue), ZINC67913374 (D, magenta), α-mangostin (E, purple), and ZINC8793749 (F, red). PC1 and PC2correspond to the dominant collective motions derived from the covariance matrix of Cα atomic fluctuations .

To further investigate the essential collective motions of hPARP1 and evaluate the ligand-induced conformational dynamics, PCA was performed on the MD trajectories of the apo and ligand-bound complexes. As seen in Fig. 4, the PCA projection along the first 2 PCs (PC1 and PC2) reflects the dominant large-scale motions sampled during simulation, where a more compact and clustered distribution indicates higher conformational stability/rigidity, whereas a broader and multi-region distribution suggests increased flexibility and multiple conformational states.

The apo hPARP1 system (Fig. S4) exhibited a widely dispersed distribution across PC space, indicating substantial conformational freedom and large-scale intrinsic motions in the absence of ligand stabilization. This broad sampling suggests that the catalytic region of PARP1 remains highly dynamic in the unbound state, likely driven by flexible loop rearrangements and domain breathing motions that contribute to an unstable binding pocket environment.

Among the ligand-bound systems, olaparib (Fig. 4A) showed an expanded and curved PCA distribution, indicating that the complex samples multiple conformational substates rather than remaining confined to a single basin. This behavior suggests that olaparib stabilizes key active site interactions while maintaining a degree of conformational adaptability, consistent with its known binding mechanism as a clinically effective PARP inhibitor. In contrast, the ZINC49069486 (Fig. 4B) and ZINC67913374 (Fig. 4D) complexes displayed notably compact PCA clusters, reflecting reduced global conformational transitions and strong restriction of collective protein motions. This indicates that both ligands impose higher structural confinement on the catalytic domain, supporting stable binding modes and enhanced rigidity of the binding pocket environment during simulation.

The ZINC98208034 (Fig. 4C) complex showed an intermediate PCA pattern, with a moderately broadened distribution suggesting partial conformational transitions between substates. This indicates that ZINC98208034 provides only moderate stabilization, allowing pronounced essential motions that may influence the persistence of binding interactions. In comparison, α-mangostin (Fig. 4E) demonstrated the most scattered and multi-cluster PCA distribution, implying extensive sampling of distinct conformational basins and high dynamic heterogeneity. Such behavior suggests weak conformational confinement and elevated structural flexibility, potentially reducing stability of the ligand-bound state. Similarly, the ZINC8793749 (Fig. 4F) complex exhibited a broad distribution with multiple conformational populations, indicating enhanced flexibility and reduced stabilization of global protein motions compared with the more compact systems.

Overall, PCA analysis demonstrates that ligand binding distinctly modulates the essential dynamics of hPARP1. The compact conformational distributions observed in the ZINC49069486 and ZINC67913374 complexes indicate enhanced structural rigidity and stabilization of collective motions relative to the apo system and other candidates, whereas α-mangostin and ZINC8793749 promote extensive conformational exploration and increased flexibility. Importantly, compact PCA clustering alone did not correlate with biological activity, as ZINC67913374 exhibited restricted conformational sampling despite lacking measurable PARP1 inhibition. In contrast, the ZINC98208034 complex exhibits a broader and more dispersed PCA distribution, suggesting pronounced conformational transitions and increased global flexibility of the protein during simulation. Although olaparib remains a validated inhibitor, its broader conformational sampling suggests a binding mechanism that stabilizes the active site while retaining controlled dynamic adaptability of the catalytic domain.

##### Energetic affinity of the hPARP1 binding complex

Supporting the previously stated results, we present the values of the energetic affinity analysis of hPARP1 complexes with 6 different ligands: olaparib, ZINC49069486, ZINC98208034, ZINC67913374, α-mangostin, and ZINC8793749. Table 5 displays the calculated binding free energy (Δ*G*_binding_, kcal/mol) obtained from the MM-PBSA method to compare the binding interface behavior, along with its contributing components: van der Waals interactions (vdW), electrostatic interactions (EEL), polar solvation energy (EPS), and nonpolar solvation energy (ENPOLAR).

**Table 5.** Energetic affinity (kcal/mol) of the candidate compounds binding to hPARP1 protein estimated by MM-PBSA calculation

Parameter	Olaparib	ZINC49069486	ZINC98208034	ZINC67913374	α-Mangostin	ZINC8793749
Δ*G*_binding_	−0.908 ± 3.985	−4.962 ± 3.469	4.206 ± 4.940	10.276 ± 5.858	4.912 ± 4.790	−4.450 ± 2.647
vdW	−56.112 ± 2.076	−48.632 ± 1.898	−48.583 ± 2.286	−79.177 ± 2.857	−55.853 ± 2.229	−27.563 ± 1.471
EEL	−9.051 ± 2.267	−9.561 ± 1.612	−10.713 ± 3.449	−19.656 ± 4.569	−12.789 ± 2.439	−5.813 ± 1.342
EPS	34.159 ± 3.809	32.329 ± 2.884	36.860 ± 5.180	68.518 ± 5.415	45.649 ± 3.561	16.28 ± 2.308
ENPOLAR	−34.742 ± 0.783	−29.924 ± 0.690	−30.698 ± 0.785	−49.701 ± 0.989	−36.562 ± 0.928	−16.437 ± 0.488

EEL and vdW, electrostatic and van der Waals contributions from molecular mechanics (MM), respectively; EPS, Poisson–Boltzmann (PB) electrostatic contribution to the solvation free energy; ENPOLAR, nonpolar contribution to the solvation free energy; Δ*G*_binding_ (kcal/mol), final estimated binding free energy calculated from the terms above

Unlike docking scores, which estimate the favorability of a ligand pose within a static binding pocket, MM-PBSA binding free energies are derived from MD trajectories and therefore incorporate conformational sampling together with solvent-related energetic contributions. As a result, direct quantitative agreement between docking scores and MM-PBSA energies is not necessarily expected. Instead, MM-PBSA analysis provides a complementary thermodynamic assessment of binding stability and can reveal unfavorable energetic factors that are not captured by docking alone.

To minimize the influence of temporal autocorrelation between adjacent trajectory frames, MM-PBSA calculations were performed using 300 decorrelated snapshots extracted at 100-ps intervals from the final 30 ns of the production trajectories. The MM-PBSA energetic decomposition revealed that vdW interactions were the dominant favorable driving force for all hPARP1–ligand complexes, indicating that hydrophobic packing and shape complementarity within the binding cleft play a major role in stabilizing ligand binding. In contrast, EEL contributions were consistently favorable but were largely counterbalanced by the EPS energy, reflecting the energetic cost of desolvating polar/charged groups upon complex formation. Notably, the ENPOLAR contribution provided an additional stabilizing effect across all systems, supporting the importance of hydrophobic burial in the catalytic pocket environment.

Compared to the positive control olaparib (Δ*G*_binding_ = −0.908 ± 3.985 kcal/mol), which exhibits a well-balanced binding mode dominated by strong vdW packing (−56.112 ± 2.076 kcal/mol) with supportive electrostatic stabilization (EEL = −9.051 ± 2.267 kcal/mol) despite polar solvation penalties, the candidate inhibitors showed distinct energetic interaction patterns reflecting different binding behaviors within the PARP1 catalytic cleft. Using decorrelated trajectory sampling, ZINC49069486 retained the most favorable binding free energies (Δ*G*_binding_ = −4.962 ± 3.469 kcal/mol), maintaining favorable vdW-driven stabilization and moderate electrostatic contributions, suggesting a comparable hydrophobic anchoring mode in the binding pocket. In contrast, ZINC98208034 and α-mangostin exhibited unfavorable binding free energies (Δ*G*_binding_ = 4.206 ± 4.940 and 4.912 ± 4.790 kcal/mol, respectively), indicating that although they can establish substantial vdW and electrostatic contacts, their binding conformations likely expose polar groups to solvent, leading to excessive desolvation costs (high EPS) and weaker net stabilization compared with olaparib. Notably, ZINC67913374 showed the strongest vdW contribution (−79.177 ± 2.857 kcal/mol), implying deep hydrophobic insertion and tight packing; however, the extremely high polar solvation penalty (EPS = 68.518 ± 5.415 kcal/mol) suggests an energetically inefficient binding mode where solvent reorganization overwhelms the favorable interactions, resulting in the poorest overall affinity (Δ*G*_binding_ = 10.276 ± 5.858 kcal/mol). Interestingly, ZINC8793749 achieved a favorable Δ*G*_binding_ (−4.450 ± 2.647 kcal/mol) close to olaparib despite weaker vdW and electrostatic terms, mainly because it produced the lowest EPS penalty among candidates, suggesting a binding pose that minimizes unfavorable polar desolvation and may represent a more solvent-compatible interaction mode.

Overall, the recalculated MM-PBSA results obtained from decorrelated trajectory sampling preserved the original energetic ranking and mechanistic interpretation. ZINC49069486 exhibited the most favorable calculated binding free energy and an energetic profile closely resembling that of olaparib, characterized by strong vdW-driven stabilization and moderate polar solvation penalties. In contrast, ZINC67913374 remained energetically unfavorable despite its strong hydrophobic packing due to an excessive EPS contribution, reinforcing the conclusion that favorable docking interactions alone are insufficient to ensure productive PARP1 binding. Collectively, these findings support ZINC49069486 as the most promising candidate among the evaluated compounds while highlighting polar solvation penalties as a critical determinant of binding efficiency.

##### Energy contributions of the individual residues in hPARP1 complexes (per-residue decomposition)

**Fig. 5.**
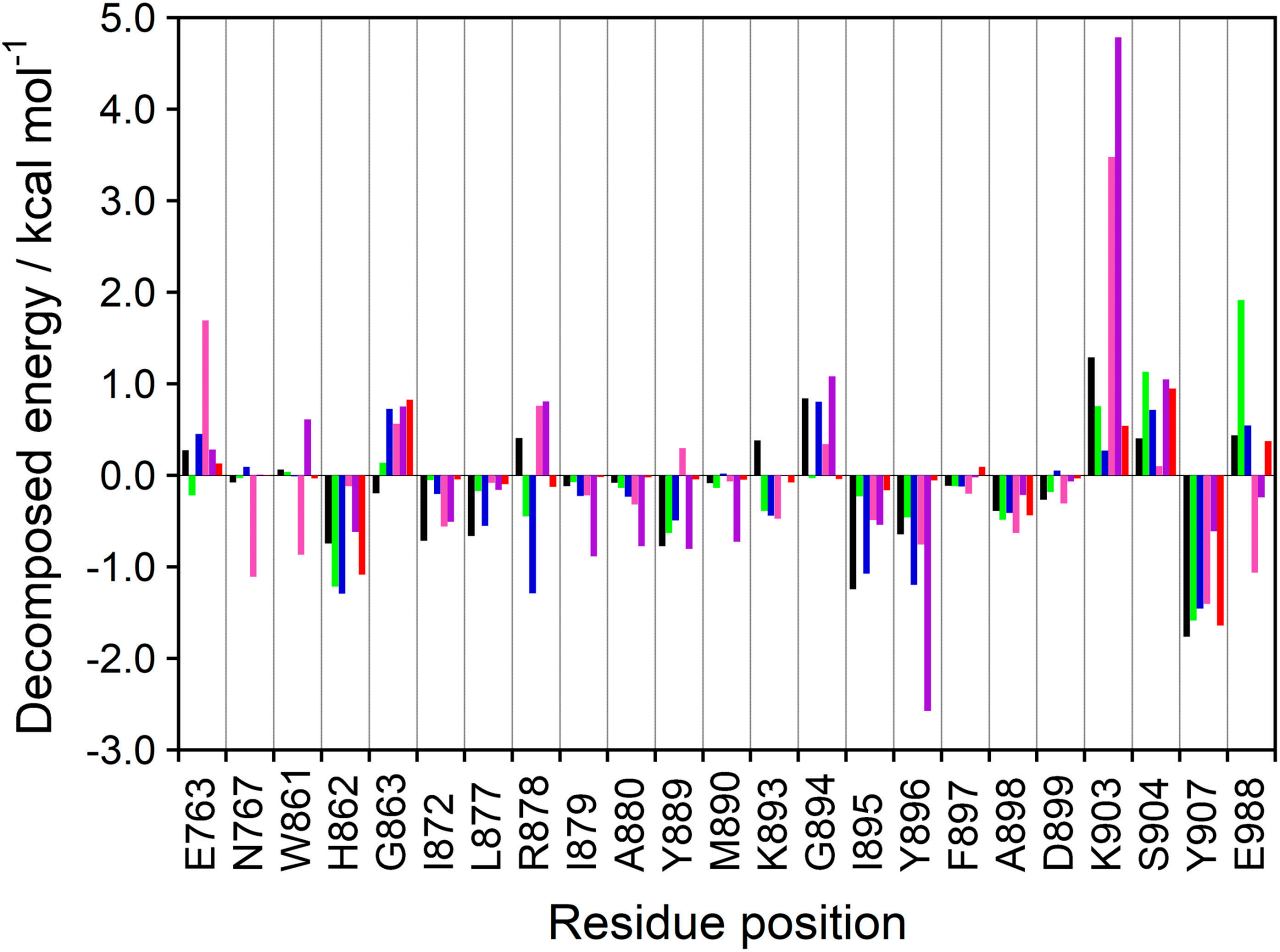
Per-residue free energy decomposition at the binding region of hPARP1–ligand bound systems. The complex systems composed of olaparib (black), ZINC49069486 (green), ZINC98208034 (blue), ZINC67913374 (magenta), ɑ-mangostin (purple), and ZINC8793749 (red) during Prod-MD simulations. All values were given in kcal/mol.

To further elucidate the residue-level determinants governing ligand stabilization within the catalytic pocket of hPARP1, per-residue free energy decomposition analysis was performed from MM-PBSA trajectories (Fig. 5 and Fig. S5). The decomposition profiles (ETOT) indicate that binding energetics are not uniformly distributed across the pocket but are dominated by a limited number of key hotspot residues within the inhibitor-binding cleft. Negative energy contributions represent favorable stabilizing interactions (e.g., hydrophobic packing, π-stacking, and electrostatic complementarity), whereas positive contributions reflect destabilizing penalties associated with steric strain, desolvation costs, or unfavorable electrostatic effects. Across all complexes, strong stabilizing contributions were consistently observed at H862 and Y907, highlighting their conserved role in anchoring ligand binding, while K903 and S904 frequently showed positive destabilizing contributions, suggesting energetic penalties at the pocket rim that influence overall complex stability and correlate with the global MM-PBSA Δ*G*_binding_ trends.

Olaparib exhibited a favorable energetic profile characterized by cooperative stabilization from several key catalytic pocket residues, particularly Y907, I895, Y889, H862, and Y896, whereas K903 and G894 contributed unfavorable energetic penalties. This interaction pattern was consistent with its overall favorable MM-PBSA binding free energy and reflected the well-established binding architecture of clinically validated PARP1 inhibitors. Notably, the dominant contributions from aromatic residues within the catalytic cleft suggest that hydrophobic packing and π-associated interactions play important roles in maintaining stable olaparib binding throughout the simulation.

Similarly, ZINC49069486 retained the same core stabilizing hotspots, with Y907 and H862 providing the strongest favorable contributions, supplemented by additional stabilization from R878, Y889, and A898. Although unfavorable contributions were observed at E988 and S904, these penalties were outweighed by the favorable interactions within the catalytic pocket, resulting in the most favorable overall binding free energy among the evaluated compounds. The preservation of key olaparib-associated interaction hotspots, together with its superior MM-PBSA profile, stable conformational behavior observed during MD simulations, potent nanomolar enzymatic inhibition, and selective synthetic lethality in BRCA1-deficient cells, collectively supports ZINC49069486 as the most promising PARP1 lead identified in this study.

ZINC8793749 also exhibited favorable MM-PBSA binding free energy (Δ*G*_binding_ = −4.450 ± 2.647 kcal/mol) primarily driven by stabilizing interactions with H862 and Y907 and accompanied by a comparatively lower polar solvation penalty (EPS = 16.28 ± 2.308 kcal/mol) than several other candidates. Nevertheless, the energetic stabilization was supported by a more limited network of favorable hotspot residues relative to olaparib and ZINC49069486, consistent with its moderate enzymatic inhibition and inability to induce a synthetic lethality response in BRCA1-deficient cells.

In contrast, ZINC98208034 displayed several favorable energetic contributions from H862, R878, Y896, I895, and Y907; however, these interactions were counterbalanced by unfavorable terms from G863, G894, S904, and E988 together with a substantial polar solvation penalty. As a results, the overall binding free energy remained unfavorable (Δ*G*_binding_ = 4.206 ± 4.940 kcal/mol), consistent with its moderate biochemical inhibition but lack of detectable synthetic lethality. ZINC67913374 showed strong local stabilization at Y907, N767, E988, and W861, and maintained favorable docking interactions within the canonical PARP1 pocket. However, these favorable contacts were overwhelmed by pronounced unfavorable energetic penalties, particularly at K903 and E763, consistent with the highest polar solvation contribution among all evaluated compounds. Consequently, ZINC67913374 displayed the most unfavorable binding free energy (Δ*G*_binding_ = 10.276 ± 5.858 kcal/mol), providing a thermodynamic explanation for its complete lack of inhibitory activity despite favorable docking and relatively stable MD behavior. Likewise, ɑ-mangostin displayed strong favorable interactions with Y896 and several hydrophobic residues, including I879, A880, Y889, and M890, resulting in an overall unfavorable binding free energy (Δ*G*_binding_ = 4.912 ± 4.790 kcal/mol) that agrees with its poor PARP1 inhibitory activity.

Collectively, the per-residue decomposition analysis identified H862 and Y907 as the most conserved favorable energetic hotspots across the PARP1–ligand complexes, highlighting their central role in stabilizing ligand binding within the catalytic pocket. Additional favorable contributions were frequently observed from L877, Y889, I895, Y896, and A898, indicating the importance of hydrophobic and aromatic interactions in maintaining productive binding. In contrast, K903 consistently emerged as the dominant unfavorable hotspot, generating substantial energetic penalties that were particularly pronounced in ZINC67913374 and ɑ-mangostin. Importantly, the residue-level energetic patterns demonstrate that favorable docking poses, stable MD conformations, or strong local hotspot interactions alone are insufficient to predict inhibitory activity. Instead, effective PARP1 inhibition requires a balanced energetic profile in which favorable pocket interactions are not outweighed by excessive polar solvation costs or unfavorable hotspot penalties. This mechanistic framework explains why ZINC49069486 and olaparib retained favorable overall binding energetics and biological activity, whereas ZINC67913374 remained inactive despite exhibiting apparently favorable docking and structural stabilization characteristics. Figure S6 shows the final conformations of the potential binding modes in each ligand bound complex Prod-MD simulations with hydrogen bond-forming residues shown in stick representation.

## Discussion

This study aimed to validate the predictive robustness and generalizability of the PARP1pred machine leaning model [22] by transitioning from computational predictions to experimental validation. Building on our previous model development, we extended the application of PARP1pred to evaluate 6 compounds, comprising the reference inhibitor olaparib and 5 candidate molecules deliberately excluded from the original training dataset to assess model performance in unseen chemical space. Among these, olaparib served as the FDA-approved positive control and was included in the original PARP1pred dataset, whereas ZINC49069486, ZINC98208034, and ZINC8793749 have been reported as PARP1-targeting compounds in the ChEMBL database [45]. ZINC67913374 was previously proposed as a PARP1 inhibitor based on molecular docking and MD analyses [18], while ɑ-mangostin, for which PARP1 is not a known target, was used as a negative control [24,25]. Through this design, we sought not only to test the predictive utility of PARP1pred for discovering active compounds beyond its original dataset but also to integrate molecular docking and MD simulations with biochemical and cellular validation in order to define the structural basis of inhibitor potency, binding stability, and PARP1-targeted activity.

Among the screened candidates, ZINC49069486 exhibited the overall inhibitory and mechanistic profile most comparable to olaparib, as supported by its biochemical potency, cellular sensitivity, favorable MM-PBSA binding energetics, conserved hotspot interactions, and stable MD behavior. Its RMSF profile remained moderate across the catalytic region without pronounced instability, indicating balanced stabilization of the PARP1 binding pocket and sustained ligand retention during simulation. PCA analysis further showed that the ZINC49069486-bound complex occupied a relatively compact conformational basin, indicating restricted large-scale motions and stable conformational sampling throughout the simulation. Although the PCA distribution did not completely overlap with that of olaparib, both systems exhibited confined conformational behavior relative to several other candidate compounds. Consistent with the MM-PBSA calculations, ZINC49069486 remained one of the most energetically favorable candidate complexes and showed the closest overall energetic profile to olaparib among the evaluated compounds. In agreement with these dynamic features, both complexes yielded favorable MM-PBSA binding free energies (Δ*G*_binding_ = −0.908 ± 3.985 kcal/mol for olaparib and −4.962 ± 3.469 kcal/mol for ZINC49069486), which is consistent with their strong biochemical PARP1 inhibitory activity in the assay. At the residue level, ZINC49069486 retained the key stabilizing hotspot pattern observed for olaparib, particularly through favorable contributions from H862 and Y907, together with additional support from Y889, Y896, and A898. Importantly, this complex showed a more balanced energetic distribution around the pocket rim than weaker candidates, indicating that productive hotspot anchoring rather than pocket occupancy alone underlies its strong inhibitory activity. In contrast, ɑ-mangostin displayed positive Δ*G*_binding_ values, indicating thermodynamically unfavorable binding, which aligns with their limited experimental activity. Energetically, binding in both systems was dominated by favorable vdW interactions, with ZINC49069486 showing a substantial vdW contribution (−48.632 ± 1.898 kcal/mol) comparable to that of olaparib (−56.112 ± 2.076 kcal/mol), supporting efficient hydrophobic packing within the catalytic pocket. Collectively, these results indicate that ZINC49069486 reproduces the key structural and energetic features of the reference inhibitor and therefore represents the most convincing lead candidate among the screened compounds.

Interestingly, while ZINC49069486 exhibits a narrower and more rigid RMSD distribution alongside superior biochemical inhibition (IC_50_ = 0.003 ± 0.001 μM) compared to olaparib (IC_50_ = 0.012 ± 0.001 μM), this catalytic superiority did not directly reflect the cellular sensitivity phenotypes. In both WT and *BRCA1^AID/AID^* cell lines, olaparib exhibited a 2- to 3-fold lower IC_50_ for cytotoxicity. This phenotypic divergence highlights that intracellular cytotoxic efficacy depends on factors beyond competitive binding at the catalytic pocket. As demonstrated by Langelier et al. [46], clinical PARPi efficacy is profoundly driven by PARP trapping, the persistent retention of inhibitor-bound PARP at DNA damage sites via both catalytic and allosteric mechanisms. Olaparib is an efficient trapper, as its potency and binding attributes lack allosteric pro-release effects [47]. In contrast, the constrained conformational rigidity observed in the RMSD and PCA profiles of the ZINC49069486-bound catalytic domain may restrict the structural flexibility necessary to trigger the allosteric structural changes required for persistent DNA trapping. Consequently, although ZINC49069486 is a more effective catalytic inhibitor in the biochemical assay, its lower cellular potency suggests a reduced trapping efficiency relative to olaparib.

The experimental results for ZINC67913374 provide a critical reassessment of its proposed PARP1 potential. Although a previous study by Li et al. [18] identified this compound as a promising candidate based on favorable docking and a highly negative reported binding free energy (Δ*G*_binding_ = −177.28 kJ/mol), our in vitro biochemical and cell-based assays did not support that prediction, as ZINC67913374 showed negligible PARP1 inhibition (IC_50_ > 100 μM) and no synthetic lethality in BRCA1-deficient cells. This discrepancy is clarified by the present integrated analysis. Although the compound retained a favorable docking score (−9.8 kcal/mol), MM-PBSA yielded a positive and unfavorable binding free energy (Δ*G*_binding_ = 10.276 ± 5.858 kcal/mol), consistent with its poor experimental performance. Notably, these findings reveal a clear divergence between structural rigidification and thermodynamic favorability, and despite producing among the lowest RMSF values across much of the catalytic region, indicating reduced local mobility, this apparent rigidification did not translate into productive binding or biological efficacy. Energetically, this complex was characterized by strong vdW and nonpolar contributions, but these favorable terms were outweighed by a large polar solvation penalty (EPS = 68.518 ± 5.415 kcal/mol). Together with its broader conformational heterogeneity during MD-based analyses, these results explain why an apparently strong docking hit can fail to achieve stable thermodynamic binding and measurable PARP1-targeted activity in experimental systems.

Importantly, the contrasting outcomes obtained from docking, MM-PBSA, and experimental analyses for ZINC67913374 illustrate the fundamentally different information provided by these approaches. While molecular docking evaluates the geometric compatibility of a ligand within a largely static binding pocket and is useful for identifying plausible binding poses, MM-PBSA further incorporates conformational sampling and solvent-related energetic contributions derived from MD simulations. Consequently, favorable docking scores do not necessarily translate into favorable binding free energies or measurable biological activity. The ZINC67913374 complex represents a clear example of this phenomenon, exhibiting favorable pocket occupancy and a relatively strong docking score, yet an unfavorable MM-PBSA binding free energy driven primarily by a substantial polar solvation penalty. This energetic behavior was consistent with its negligible PARP1 inhibition in the biochemical assay and the absence of synthetic lethality in BRCA1-deficient cells. Collectively, these findings emphasize that docking scores should be interpreted primarily as indicators of potential binding modes rather than quantitative measures of binding affinity or inhibitor potency, highlighting the importance of integrating dynamic, energetic, and experimental validation when evaluating candidate PARP1 inhibitors.

Despite sharing quinazolinone-related structural features and showing measurable biochemical activity, ZINC98208034 and ZINC8793749 did not translate into a robust PARP1-targeted cellular phenotype. ZINC98208034 exhibited moderate PARP1 inhibition in the biochemical assay (IC_50_ = 12.238 ± 1.808 μM) but failed to induce clear synthetic lethality in *BRCA1^AID/AID^* cells, consistent with our previous report classifying this compound as an intermediate inhibitor [22] and with the study of Lindgren et al. [48], which suggested preferential selectivity toward PARP3 rather than PARP1. Structurally, its bimodal RMSD distribution, with peaks at ~2.2 and ~2.7 Å, indicates that the ligand was not maintained in a single well-confined binding arrangement, likely due to the flexible aliphatic C–C linker connecting its heterocyclic core to the terminal aromatic group. This interpretation is further supported by its elevated RMSF in the ~700- to 780-residue region [49,50] and by residue-level decomposition showing that otherwise favorable hotspot contributions at H862, R878, I895, Y896, and Y907 were offset by unfavorable penalties at G863, G894, S904, and E988. As a result, the relatively high polar solvation penalty (EPS = 36.860 ± 5.180 kcal/mol) outweighed favorable intermolecular interactions and yielded an unfavorable Δ*G*_binding_ of 4.206 ± 4.940 kcal/mol. In contrast, ZINC8793749 produced a favorable net MM-PBSA binding free energy (Δ*G*_binding_ = −4.450 ± 2.647 kcal/mol), but this result appeared to arise mainly from its relatively low polar solvation penalty rather than from strong binding site stabilization. Consistent with this, ZINC8793749 showed only moderate enzymatic inhibition (IC_50_ = 15.791 ± 4.053 μM) and failed to induce synthetic lethality in BRCA1-deficient cells, while docking, RMSD, RMSF, PCA, and per-residue decomposition all indicated a weakly anchored binding mode with limited favorable hotspot support, primarily centered on H862 and Y907. Thus, although both compounds can engage the PARP1 catalytic pocket, ZINC98208034 appears to be limited by scaffold flexibility and incomplete energetic complementarity, whereas ZINC8793749 lacks the broader cooperative interaction network required for persistent and biologically productive PARP1 inhibition.

Per-residue free energy decomposition provided a unifying cross-system explanation for the differences in biochemical activity, cellular response, and computational binding behavior among the evaluated hPARP1 complexes. Across all systems, H862 and Y907 emerged as the most conserved favorable hotspots, indicating that these residues serve as the principal anchoring points required for productive ligand engagement within the catalytic pocket. Additional stabilizing contributions were frequently observed from Y889, I895, Y896, and A898, which together define a supportive hydrophobic–aromatic framework for ligand confinement. In contrast, K903 and S904 consistently contributed unfavorable positive energies in multiple complexes, identifying the pocket rim as a recurrent source of energetic penalty that can offset otherwise favorable interactions. Notably, the most active inhibitors, olaparib and ZINC49069486, exhibited the most balanced residue-level interaction patterns, combining strong anchoring at H862/Y907 with broader stabilizing support from neighboring residues and comparatively reduced destabilizing penalties. By contrast, experimentally weak or inactive compounds either relied on a narrower interaction pattern, as observed for ZINC8793749, or showed strong local stabilization that was outweighed by unfavorable energetic terms, as seen for ZINC98208034, ZINC67913374, and α-mangostin. Collectively, these results indicate that productive PARP1 inhibition depends not only on pocket occupancy or local rigidification but also on a balanced residue-level interaction network that maintains catalytic site stabilization while minimizing energetic penalties at the solvent-exposed pocket rim.

While several studies have integrated ML with molecular docking and dynamics to identify PARP1 inhibitors, many lack both in vitro validation and accessible platforms for noncomputational scientists [21,51–53]. Aldakheel et al. [21] and Shahab et al. [51] both employed random forest models, trained on BindingDB and ChEMBL datasets, respectively, to prioritize candidates for subsequent MD simulations and MM-PBSA/GBSA (molecular mechanics Poisson–Boltzmann surface area/generalized Born surface area) analysis. Similarly, Caba et al. [52] utilized a support vector machine (SVM)-based regressor and Graph Neural Networks (DeepCoy) for structure-based virtual screening, while Nguyen et al. [53] combined diffusion-based generative models with deep-learning (DL) filters to navigate the ZINC20 chemical space. To bridge the gap between complex informatics and public accessibility, Ai et al. [54] developed a webserver for classifying activity across 4 PARP isoforms, including PARP1. However, despite these computational advancements, experimental confirmation remains scarce. Notable exceptions include the work of Truong et al. [55] and Bacha et al. [56], who utilized Deep Docking to screen 1.6 billion compounds, ultimately validating their artificial intelligence-prioritized leads through chemical synthesis, in vitro*,* and in vivo models. While operating at a different scale, our study follows the same rigorous principles by transitioning from ML-driven prioritization (PARP1pred) and MD simulations to empirical biochemical and cellular validation. Consequently, our pipeline reinforces the necessity of moving beyond in silico predictions to ensure therapeutic relevance in a biological context.

Altogether, the contrasting outcomes of ZINC67913374 and ZINC49069486 underscore the predictive robustness and practical utility of the PARP1pred model. Although conventional structure-based screening previously highlighted ZINC67913374 as a promising hit based on favorable docking and apparent pocket occupancy [18], our integrated validation showed that this compound was biologically inactive, with negligible PARP1 inhibition, no synthetic lethality in BRCA1-deficient cells, and an unfavorable MM-PBSA binding free energy. In contrast, ZINC49069486, which was prioritized by PARP1pred, consistently demonstrated the expected profile of a true PARP1 inhibitor, including sub-nanomolar biochemical potency, selective cellular hypersensitivity in BRCA1-deficient cells, stable docking within the catalytic pocket, olaparib-like dynamic behavior during MD simulations, and a favorable Δ*G*_binding_. These findings indicate that pocket occupancy or docking rank alone is insufficient to identify functional inhibitors, whereas PARP1pred more effectively captures the substructural features required for productive PARP1 engagement. Moreover, our results support a hierarchical discovery framework in which ML-driven screening serves as a high-fidelity primary filter, followed by experimental validation, and then MD/MM-PBSA analyses to resolve binding stability and energetic barriers. This layered approach is particularly important for compounds such as ZINC98208034 and ZINC67913374, where apparently acceptable docking poses were not supported by favorable thermodynamics or experimental activity. Furthermore, decomposition of vdW, EEL, EPS, and ENPOLAR terms provides medicinal chemists with a precise map for lead optimization by pinpointing whether ligand binding is limited by weak packing, insufficient electrostatic complementarity, or excessive polar solvation penalties. Although long-timescale MD simulations remain computationally intensive and unsuitable for large-scale initial screening, the PARP1pred webserver facilitates rapid and accessible screening for prioritizing candidates, thereby helping both computational and experimental researchers, including natural product investigators, to focus resources on compounds with a higher probability of genuine PARP1 inhibitory activity.

An additional consideration is that the molecular docking scores and MM-PBSA binding free energies reported in this study should not be interpreted as experimentally determined binding affinities. Docking analysis provides an estimate of ligand compatibility within a static binding pocket, whereas MM-PBSA evaluates the energetic stability of protein–ligand complexes after conformational sampling during MD simulations. Although these approaches provide valuable mechanistic insights into ligand recognition and binding behavior, they do not constitute direct measurements of dissociation constants (*K*_d_). Therefore, experimental biophysical techniques such as surface plasmon resonance (SPR), biolayer interferometry (BLI), microscale thermophoresis (MST), or isothermal titration calorimetry (ITC) would be required to quantitatively determine binding affinities and further validate the interaction strengths of the identified candidates.

Despite the effectiveness of the present hierarchical workflow for identifying and validating PARP1 inhibitory candidates, several limitations and future opportunities should be acknowledged. At present, the pipeline is primarily optimized for early-stage hit prioritization and does not yet incorporate absorption, distribution, metabolism, excretion, and toxicity (ADMET) prediction, which will be essential for assessing the developability and clinical feasibility of candidate compounds. This issue is particularly relevant for natural product-like scaffolds, which may exhibit constraints in solubility, membrane permeability, metabolic stability, or off-target toxicity despite promising biochemical or cellular activity. In addition, although PARP1pred effectively prioritizes compounds with PARP1 inhibitory potential, its current design does not explicitly resolve subtle isoform-selective interactions within the PARP family, especially between closely related targets such as PARP1 and PARP2. Furthermore, while current computational constraints preclude the immediate implementation of advanced allosteric modeling, we propose that future iterations of the PARP1pred framework should incorporate an in silico benchmarking module. This module would ideally utilize distinct trapping typologies, such as highly efficient trappers (e.g., EB-47) and release-promoting candidates (e.g., veliparib) [46], to better align computational predictions with clinical outcomes. Addressing these limitations through integrated ADMET filtering, automated docking, selectivity-focused modules, and trapping-tier categorization would substantially improve the translational value of the webserver as a near-term development goal. Beyond these refinements, the PARP1pred framework could be further extended to explore a much broader chemical universe through integration with emerging large-scale and generative computational platforms. In particular, incorporation of Deep Docking [55,56] would enable efficient screening of ultra-large virtual libraries containing billions of compounds, thereby increasing the likelihood of identifying novel PARP1-binding scaffolds beyond conventional natural product and traditional databases. Likewise, generative design strategies such as ClickGen [57] could support de novo development of PARP1 inhibitors by combining reinforcement learning with modular click-chemistry rules to generate structurally diverse and synthetically accessible candidates. Collectively, these advances would shift the current workflow from compound prioritization alone toward a more comprehensive discovery platform capable of both screening existing molecules and designing new lead structures. Although implementation of such DL-driven approaches remains dependent on access to high-performance computational infrastructure, continued improvements in affordability and accessibility may ultimately enable PARP1pred to evolve into a scalable platform for accelerated discovery of potent, selective, and pharmacologically optimized PARP1 inhibitors.

## Conclusion

This study validated a hierarchical drug discovery pipeline by integrating the PARP1pred ML model with structure-based simulations and in vitro experiments. Our findings demonstrate that PARP1pred accurately categorized bioactivity in unseen chemical space, successfully identifying ZINC49069486 as a potent lead with a sub-micromolar IC_50_ of 3 nM and the ability to induce synthetic lethality in BRCA1-deficient cells. Crucially, the model correctly classified ZINC67913374 as inactive, correcting previous structure-based predictions that overlooked substantial polar solvation penalties and conformational heterogeneity revealed by our MM-PBSA and MD analyses. While the pipeline effectively distinguished between high-potency leads and moderate hits like ZINC98208034 and ZINC8793749, current limitations include a lack of ADMET filtering, isoform-selectivity prediction, and explicit modeling of PARP trapping mechanisms. Nevertheless, the availability of the PARP1pred webserver combined with dynamic energetic validation provides a robust framework for prioritizing pharmacologically relevant compounds from diverse chemical databases, thereby conserving experimental resources in the early stages of drug discovery. The present findings further demonstrate that docking-derived pocket occupancy alone is insufficient to reliably identify functional PARP1 inhibitors and should be complemented by energetic and experimental validation.

## Data Availability

All data needed to evaluate this study are available within the article and its Supplementary Materials. Additional data supporting the findings of this study are available from the corresponding authors upon reasonable request.
