# Supplementary material for: Experimental and Mechanistic Validation of PARP1pred for Identifying Potent Leads
Source: Comput Struct Biotechnol J. 2026 Jul 7;35(1):0152. doi: 10.34133/csbj.0152 (PMC13338561; doi:10.34133/csbj.0152)
Supplement: Supplementary 1 — Figs. S1 to S6 Tables S1 to S3 [file csbj.0152.f1.zip › Revised_Supporting-Information_26.05.2026.docx]

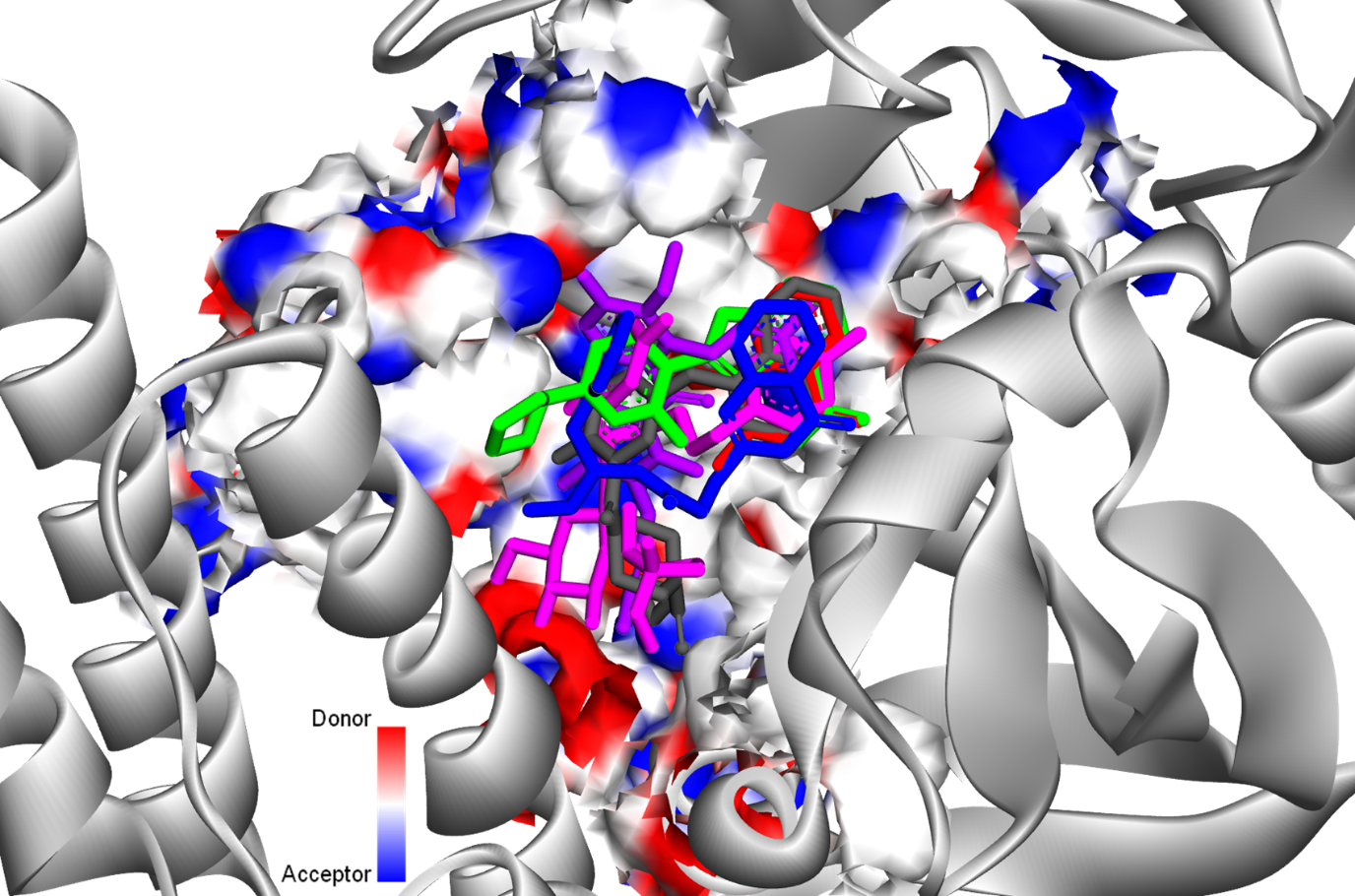


# Fig. S1. Docking models of olaparib (black), ZINC49069486 (green), ZINC98208034 (blue), ZINC67913374 (pink), α-mangostin (purple), and ZINC8793749 (red) in binding pocket region of the hPARP1 (PDB ID: 4UND) protein. The potential site of the hPARP1 is divided into the surface display style that is colored by hydrogen bond donor/acceptor in blue-white-red spectrum range.


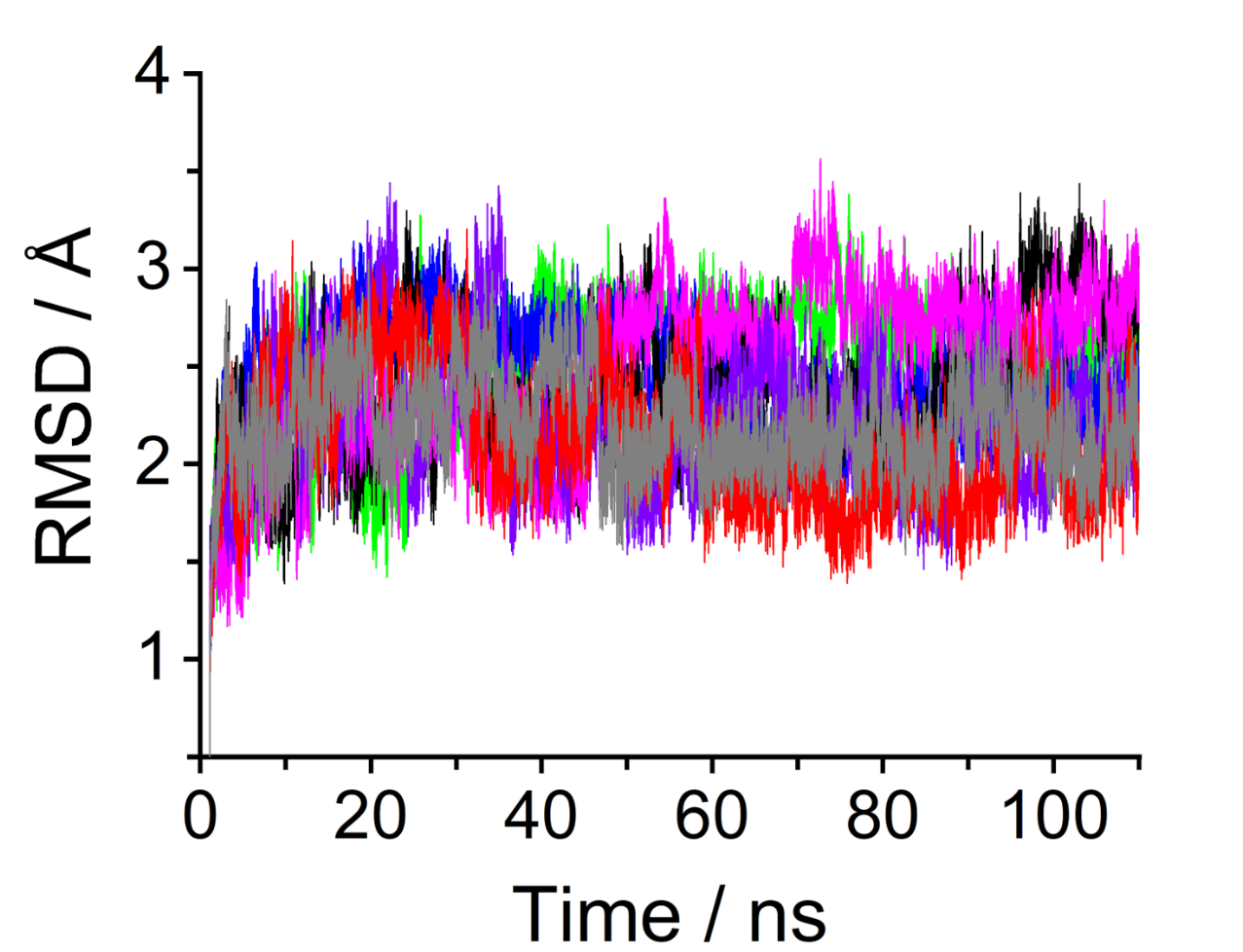


# Fig. S2. Comparison of RMSD plots of of all Cα-atoms of the hPARP1 complex system; olaparib (black), ZINC49069486 (green), ZINC98208034 (blue), ZINC67913374 (magenta), α-mangostin (purple) and ZINC8793749 (red) analyzed during Prod-phase MD simulations.


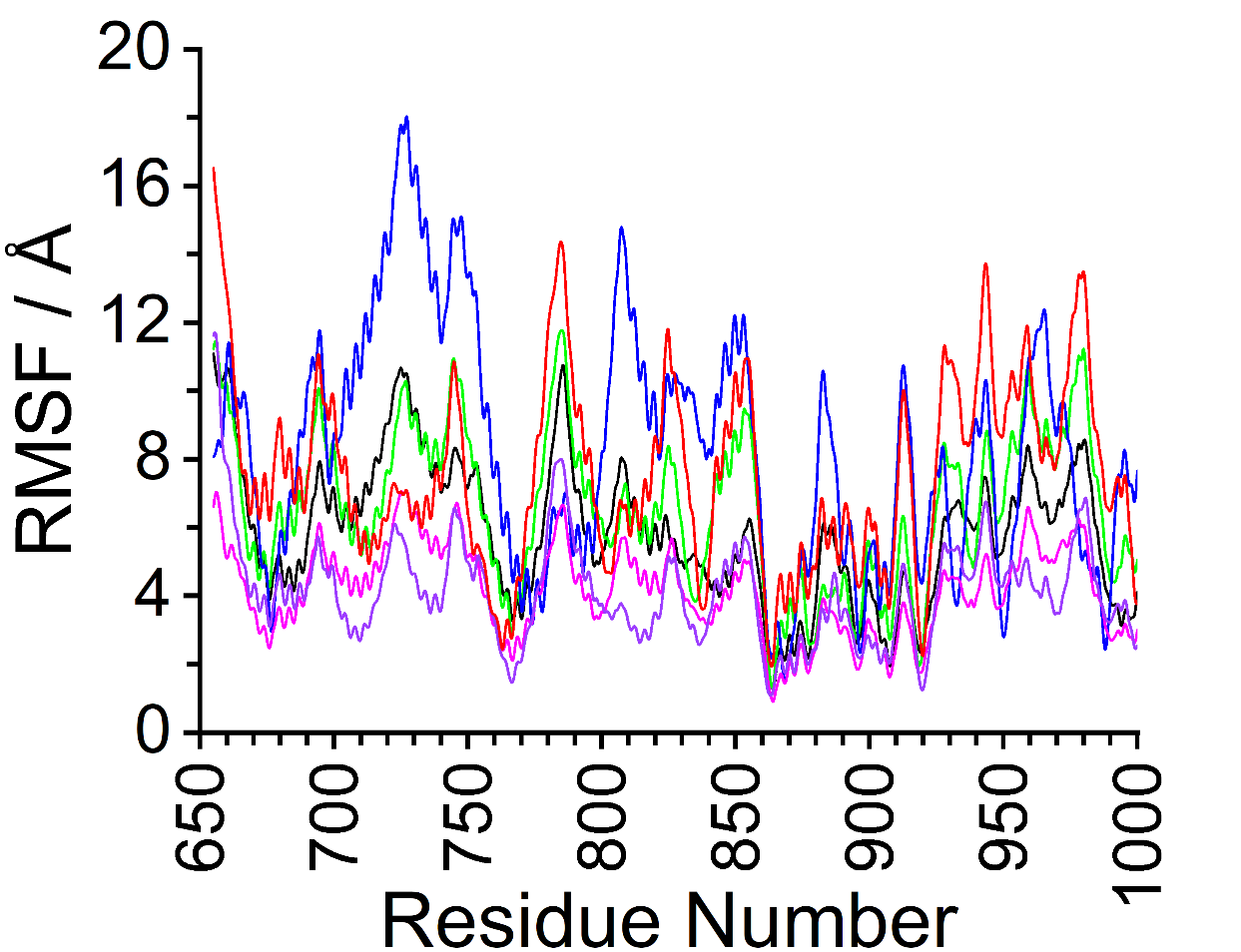


# Fig. S3. Full RMSF profiles in each complex of Cα-atom of the hPARP1 protein (residue 655-1010) of Olaparib (black-solid line), ZINC49069486 (green-solid line), ZINC98208034 (blue-solid line), ZINC67913374 (magenta-solid line), ɑ-mangostin (purple-solid line), and ZINC8793749 (red-solid line), which represents the residue number according to the X-ray data (PDB ID: 4UND).


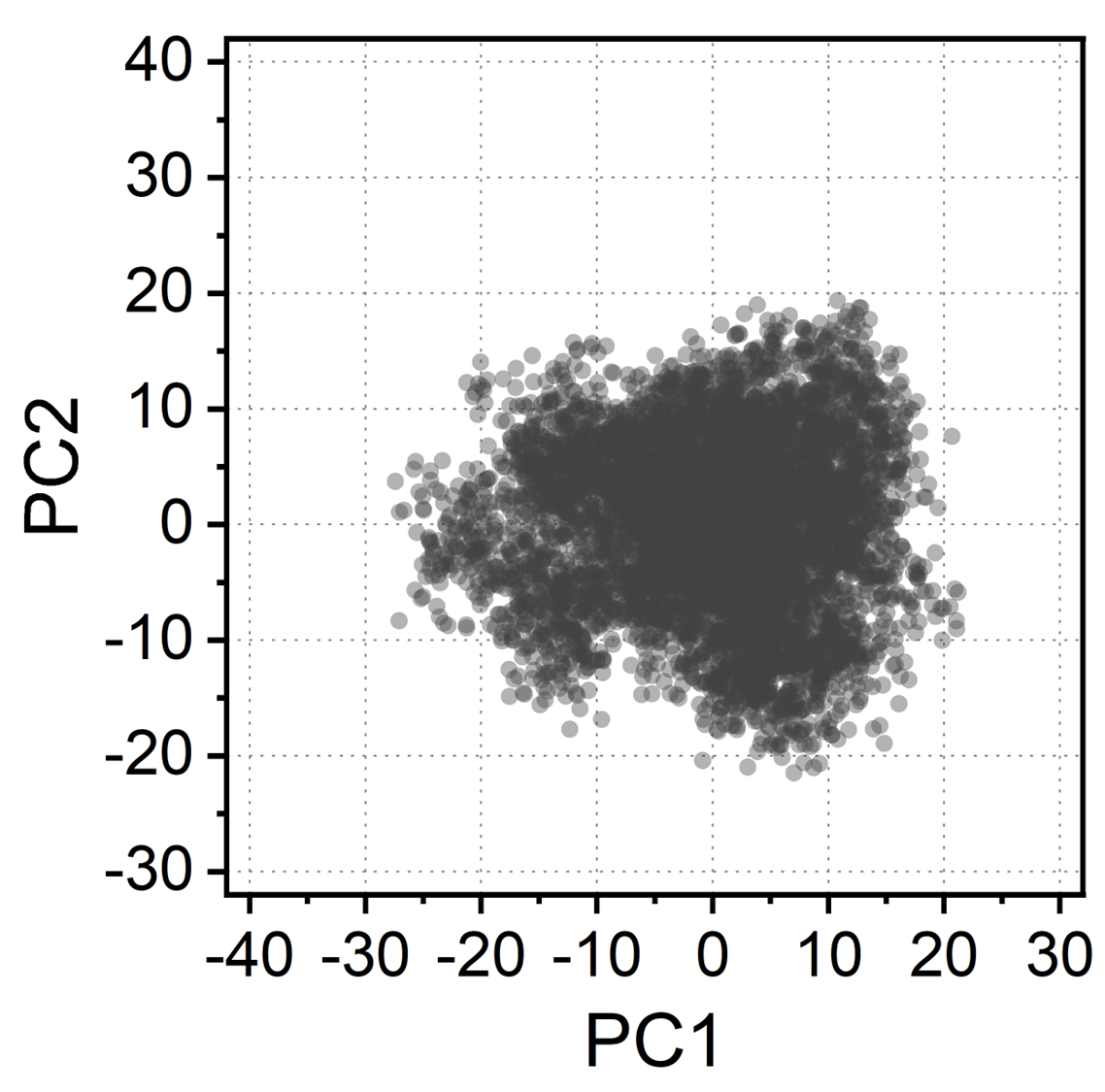


# Fig. S4. PCA projection of the motion of Cα-atom of the hPARP1 Apo system. PCA profiles were obtained by plotting the first two principal components (PC1 and PC2) in the conformational space, respectively, which represent a covariance matrix after elimination of eigenvectors.


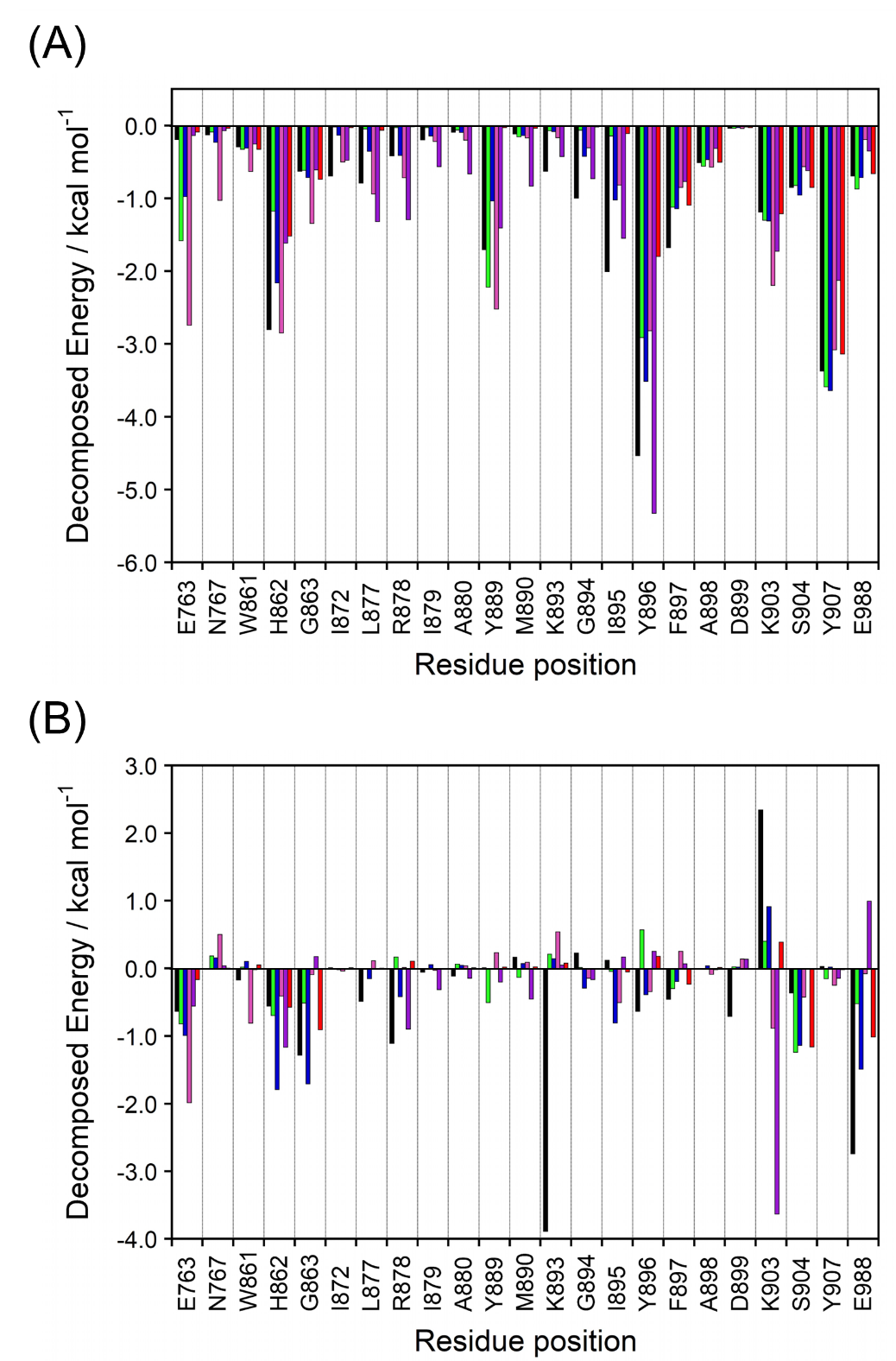


# Fig. S5. Energy decomposition analysis of hPARP1 protein in complex with olaparib (black), ZINC49069486 (green), ZINC98208034 (blue), ZINC67913374 (magenta), α-mangostin (purple) and ZINC8793749 (red). Per-residue decomposed energy of (A) vdW interaction, and (B) electrostatic on the key hotspots of the hPARP1 regions for binding. All values were given in kcal/mol.


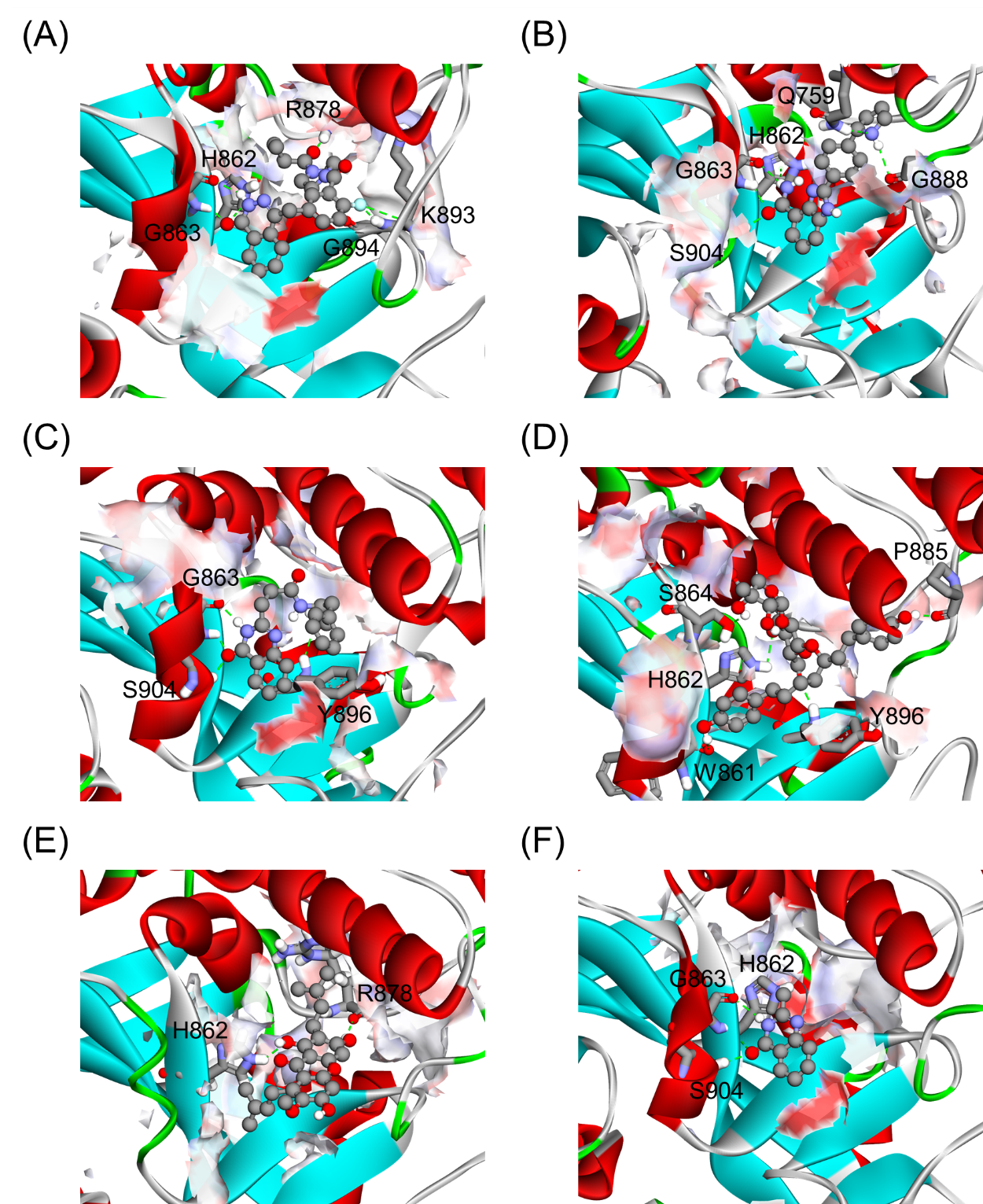


# Fig. S6. Structural details of the hPARP1 binding interaction with (A) olaparib, (B) ZINC49069486, (C) ZINC98208034, (D) ZINC67913374, (E) α-mangostin and (F) ZINC8793749 at the binding interface obtained from the Prod phase MD simulations. The secondary structures of hPARP1 are portrayed as ribbons style, while each ligand is represented in balls and sticks. Each interacting amino acid residue is highlighted in black-color labeled. The hydrogen bonds are represented in a green dashed line.
